# Identifying metabolic enzymes with multiple types of association evidence

**DOI:** 10.1186/1471-2105-7-177

**Published:** 2006-03-29

**Authors:** Peter Kharchenko, Lifeng Chen, Yoav Freund, Dennis Vitkup, George M Church

**Affiliations:** 1 Department of Genetics, New Research Building (NRB) Room 238, 77 Ave. Louis Pasteur, Harvard Medical School, Boston, MA 02115, USA; 2 Center for Computational Biology and Bioinformatics, Department of Biomedical Informatics, Columbia University, 1150 St. Nicholas Ave., New York, NY 10032, USA; 3 Department of Computer Science and Engineering, University of California San Diego, 9500 Gilman Drive 0404, Room 4126, La Jolla, CA 92093, USA

## Abstract

**Background:**

Existing large-scale metabolic models of sequenced organisms commonly include enzymatic functions which can not be attributed to any gene in that organism. Existing computational strategies for identifying such missing genes rely primarily on sequence homology to known enzyme-encoding genes.

**Results:**

We present a novel method for identifying genes encoding for a specific metabolic function based on a local structure of metabolic network and multiple types of functional association evidence, including clustering of genes on the chromosome, similarity of phylogenetic profiles, gene expression, protein fusion events and others. Using *E. coli *and *S. cerevisiae *metabolic networks, we illustrate predictive ability of each individual type of association evidence and show that significantly better predictions can be obtained based on the combination of all data. In this way our method is able to predict 60% of enzyme-encoding genes of *E. coli *metabolism within the top 10 (out of 3551) candidates for their enzymatic function, and as a top candidate within 43% of the cases.

**Conclusion:**

We illustrate that a combination of genome context and other functional association evidence is effective in predicting genes encoding metabolic enzymes. Our approach does not rely on direct sequence homology to known enzyme-encoding genes, and can be used in conjunction with traditional homology-based metabolic reconstruction methods. The method can also be used to target orphan metabolic activities.

## Background

Comprehensive and accurate reconstruction of the metabolic networks remains an important problem for both newly sequenced and well-studied organisms [1,2]. The challenges posed by the experimental determination of the metabolic enzymes have lead to development of computational methods for metabolic reconstruction. The most common approach is to identify genes encoding a specific metabolic enzyme by establishing sequence homology to functionally characterized enzymes in other species [3]. Although such sequence homology methods have been remarkably successful overall, they fail to identify enzymes encoded by genes with poor sequence homology to known metabolic enzymes, and result in partially reconstructed metabolic networks. In some cases sufficient biological evidence exists to believe that a particular pathway is present in the organism, however genes associated with key reaction steps can not be identified. The problem of identifying genes encoding for a specific metabolic function in such partially reconstructed networks has been referred to as the "missing gene" problem [4]. The case for a missing gene can be based either on direct experimental evidence for a particular enzymatic function in the organism, or on variety of comparative and computational analysis of known metabolic pathways, biochemical constraints and environmental conditions [4,5]. We note that the problem of identifying missing genes, considered in this manuscript, is different from the traditional problem of functional gene annotation, which aims to assign function to a given gene.

Computational strategies for identifying missing metabolic genes rely on refined sequence homology analysis [2,6] and consideration of functional association evidence linking candidate genes with known enzyme-encoding genes [4]. For example, PathwayTools hole-filler developed by Green *et al*. [6], prioritizes candidates obtained from an initial sequence homology search by using, among other factors, information on whether the candidate gene is located adjacent to, or in the same transcriptional unit as known enzyme-encoding genes of related metabolic function. In some cases, strong genome context association evidence, such as clustering of genes on the chromosome, or co-occurrence of genes in phylogenetic lineages, has played a key role in identifying metabolic genes in several organisms [7-9].

An extensive set of tools has been developed to detect and catalog general pair-wise functional associations between genes based on a combination of genome context methods and other evidence, such as co-expression or protein interactions [10,11]. Combinations of heterogeneous association evidence have been used for general functional inference [12], prediction of protein complexes [13-15] and synthetic lethal interactions [16]. A recent work by Yamanishi *et al*. [17] relied on a combination of genomic, mRNA expression and localization evidence, together with information on chemical compatibility to reconstruct metabolic pathways from known metabolic enzymes. While it has been suggested that genome context associations can be used for general prediction of missing enzyme-encoding genes [4,18], methods for systematic targeting of missing genes have not been characterized. We develop a method for generating such predictions based on combination of genome context and other functional association evidence, and show that it is effective for majority of the enzymatic functions in *E. coli *and *S. cerevisiae*.

**Figure 1:**
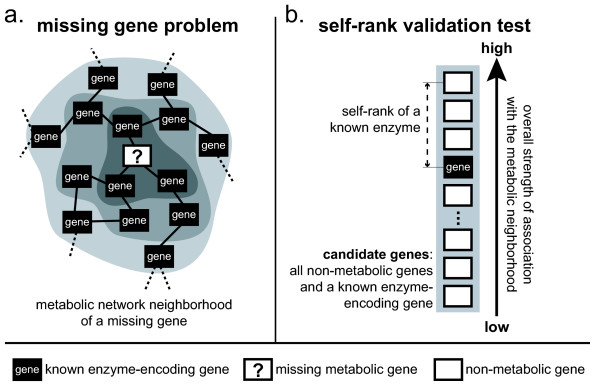
**a. **Illustration of the missing gene problem. Metabolic network neighborhood of a missing metabolic enzyme is shown. The neighborhood comprises layers with increasing radii (3 layers shown, indicated by shading). Majority of the enzyme-encoding genes in the neighborhood are known. **b. **Illustration of the self-rank validation test. Ability to predict known enzyme-encoding genes is tested by measuring its *self-rank *- the rank of a true enzyme-encoding gene in the candidate set. The candidates are ordered according to overall strength of their functional association with the metabolic network neighborhood of the enzyme. The overall association strength is a combination of layer association scores that measure strength of functional association of the candidate gene with known enzyme-encoding genes in a single layer of the metabolic neighborhood (3 layers, as illustrated in a.). The candidate set contains all genes that are not already part of the metabolic network.

In an earlier study we have described a method for identifying missing enzyme-encoding genes based on gene co-expression and local structure of metabolic network [19]. The candidate genes for encoding a missing metabolic enzyme were evaluated based on the overall similarity of their expression profile with the expression of the metabolic network neighborhood of the missing enzyme (Figure 1a). The local property of gene co-expression, which formed the basis of this method, is also observed for other types of functional associations, in particular for associations established by genome context [20]. In this work we show that such approach can be extended to identify metabolic enzyme-encoding genes from a number of different types of functional association evidence, including phylogenetic profile co-occurrence, physical clustering of genes on the chromosome and protein interaction data. We note that the presented method does not rely on sequence homology to known enzymes, and its predictions are complementary to the traditional methods of metabolic reconstruction.

We illustrate the performance of each individual type of association evidence by testing how well the method is able to predict known enzyme-encoding genes of *E. coli *[2] and *S. cerevisiae *[21] metabolic models (see Methods). A set of candidate genes, containing all non-metabolic genes in an organism, is evaluated and prioritized by evaluating overall association of each candidate gene with the neighborhood of the missing metabolic enzyme (Figure 1b). The overall association is calculated by combining associations with each layer of the metabolic neighborhood. Specifically, for a missing enzyme with a metabolic neighborhood consisting of layers {*L*_1_, *L*_2_, *L*_3_}, each candidate gene *x *is evaluated by a combination of layer association scores, scoreLi
 MathType@MTEF@5@5@+=feaafiart1ev1aaatCvAUfKttLearuWrP9MDH5MBPbIqV92AaeXatLxBI9gBaebbnrfifHhDYfgasaacH8akY=wiFfYdH8Gipec8Eeeu0xXdbba9frFj0=OqFfea0dXdd9vqai=hGuQ8kuc9pgc9s8qqaq=dirpe0xb9q8qiLsFr0=vr0=vr0dc8meaabaqaciaacaGaaeqabaqabeGadaaakeaacqWGZbWCcqWGJbWycqWGVbWBcqWGYbGCcqWGLbqzdaWgaaWcbaGaemitaW0aaSbaaWqaaiabdMgaPbqabaaaleqaaaaa@3671@ (*x*), that measure the strength of functional associations of candidate gene *x *with known enzyme-encoding genes in the neighborhood layer *L*_*i *_(see Methods). The individual layer association scores are combined using one of two methods (ADT or DLR) to obtain a measure of overall association of candidate gene *x *with the metabolic network neighborhood of the missing enzyme.

To assess the performance of our method we rely on a self-rank measure, which is the rank of a known enzyme-encoding gene among the set of candidates prioritized for its own metabolic function (see Methods). We develop techniques for combining multiple types of association evidence and show that significantly better prediction performance can be achieved based on combined association evidence.

## Results

### Similarity of phylogenetic profiles

A number of earlier studies have explored using patterns of gene co-occurrence or absence in the phylogenetic lineages to infer functional association between gene pairs [22,23]. The basic premise of the method is that a function is likely to be encoded by several associated genes; therefore lineages maintaining only some of these genes will have lower evolutionary fitness. For instance, enzymes catalyzing successive steps of a linear metabolic pathway are likely to be present together in an organisms relying on that metabolic pathway, and absent together from an organisms that does not require that pathway.

A phylogenetic profile of a given gene on a set of *N*_G _genomes can be encoded as binary string of length *N*_G_, with each position marking presence (1) or absence (0) of an ortholog in the corresponding genome. Functional association between a pair of genes is assessed by the degree of similarity of their phylogenetic profiles. A number of different distance measures have been used to calculate such similarity, including Hamming string distance, mutual information and hypergeometric distribution [11,22,24,25]. We find that the performance of different distance measures is very similar (see Additional file 1). These profile similarity measures do not take into account variable degree of divergence between genomes comprising the orthology dataset. This is particularly clear in the case of Hypergeometric distribution measure [11,25], which assumes that ortholog occurrences are independently and identically distributed across the set of included genomes (see Methods).

The identity assumption would suggest that the total number of ortholog occurrences within each genome should be approximately the same, and the distribution of the number of orthologs should form a single, narrow peak around an average ortholog number. The empirical distribution (see Additional file 2), however, is quite different from the expected form, lacking a peak around the mean, and showing substantial density over almost an entire range of ortholog numbers. When the identity assumption is relaxed, profile similarity probability is described by the Extended Multivariable Hypergeometric distribution [26]. Because probability functions of this distribution have not been derived in a closed form, we developed a numerical algorithm for estimating these probabilities (see Methods).

Bias stemming from the violation of the independence assumption can be minimized by exclusion or reduction of closely related species in the ortholog occurrence dataset. We employ a method similar to previously published work [10], which reduces the bias by folding together phylogenetic branches containing closely related species, and using an ortholog occurrence pattern based on the agreement within the folded branch (see Methods).

**Figure 2:**
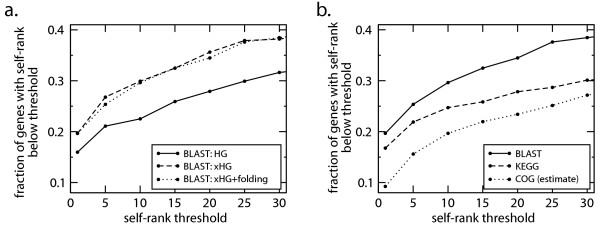
Performance of different phylogenetic profile datasets and corrections. The predictive performance of the algorithm is illustrated by showing the fraction of known enzyme-encoding genes (x axis) predicted within different self-rank thresholds (y axis). For instance, dashed performance curve in subfigure a. (BLAST:xHG) shows that 30% of the test enzymes appear within the top 10 (out of 3352) candidates for their enzymatic function. **a. **Algorithm performance in predicting known *E. coli *metabolic enzymes based on the phylogenetic profile associations with the 1^st ^layer of the metabolic network neighborhood. Performance of a regular hypergeometric distribution is shown (HG), together with extended hypergeometric (xHG) and folding (xHG+folding) corrections. The scores are calculated on the BLAST-based dataset. **b. **The self-rank performance of the 1^st ^layer phylogenetic profile score, calculated using extended hypergeometric distribution with folding is shown for BLAST-based, KEGG-based and COG orthology datasets. The performance of the COG orthology dataset is corrected for the metabolic gene coverage bias.

The effect of both corrections on the ability to predict enzyme-encoding genes in *E. coli *is illustrated by the cumulative self-rank distributions (see Methods) in Figure 2a. The extended hypergeometric distribution correction for the variable genome divergence from *E. coli *target genome (violation of the identity assumption) provides a noticeable improvement in prediction performance (8% at self-rank threshold of 50). On the other hand, the folding method correcting for variable divergence with the set of query genomes (violation of independence assumption) does not significantly improve the results.

The phylogenetic profile co-occurrence method depends on identification of orthologous genes across potentially diverse lineages. Existing investigations have used a variety of methods, including readily available *Clusters of Orthologous Groups *(COG) database [27,10,25], closest homologs [11], and best bi-directional homology pairs [24]. The results presented in our work rely on two alternative sets of orthology data. The first set comes from KEGG SSDB database [28], and includes closest homologs and best bi-directional hits as determined by the Smith and Waterman algorithm (we will refer to it as KEGG-based dataset). The second set was constructed based on results of BLAST [29] queries against a "non-redundant" set of known protein sequences maintained by NCBI (see Methods). The set also includes information on reverse BLAST searches to determine best bi-directional hits (referred to as BLAST-based dataset).

Predictive performance of different orthology datasets is compared in Figure 2b. We note that coverage of the COG orthology data is biased towards genes encoding known metabolic enzymes (see Additional file 16), and the self-rank performance of this dataset was estimated by normalizing with respect to the non-metabolic gene coverage. Figure 2b shows that profile associations calculated using BLAST-based dataset provide better predictions of enzyme-encoding genes than association based on the KEGG orthology dataset. We also find that in the case of both datasets better performance is attained when using best bi-directional homology pairs instead of closest homologs (see see Additional file 3).

As a consequence of gene duplications, metabolism contains a significant number of paralogous enzyme pairs [30]. In many cases, such enzymes continue to catalyze the same reactions (see Additional file 4). Such pairs will frequently have similar or identical orthology mappings, and their inclusion can lead to a significant bias in estimation of the predictive performance (see Additional file 5). The results presented in this work, therefore, exclude self-ranks of any metabolic enzymes that have high sequence homology to any other metabolic enzyme in the organism (see Methods).

### Co-expression of orthologous genes

The approach for identifying enzyme-encoding genes based on the similarity of mRNA expression profiles [19] can be extended to include co-expression information of orthologous genes in other organisms. Conservation of mRNA co-expression across different species has been investigated by a number of recent studies [31-34]. For example, analysis of co-expressed gene pairs between *S. cerevisiae *and *C. elegans *shows statistically significant (P value < 10^-3^) level of conservation [32]. Although the number of pairs with highly conserved co-expression is small, incorporating ortholog co-expression can provide significant improvements to the accuracy of functional predictions based on the mRNA expression data [31,32].

We find that enzyme-encoding gene predictions based on the co-expression of *E. coli *orthologs in *S. cerevisiae *(see Methods) achieve good performance on the enzymes covered by such dataset. Although *S. cerevisiae *orthologs can be identified for only 40.1% of *E. coli *metabolic genes, combining native and ortholog co-expression scores provides noticeable improvements. Combination of native and ortholog co-expression increases the fraction of metabolic enzymes predicted within the top 50 candidates from 27% to 36% (see Additional file 6). Similarly, using *E. coli *expression data improves prediction results for enzyme-encoding genes of *S. cerevisiae *metabolic network. Overall self-rank performance based on combined co-expression data is included in Figure 4.

### Clustering of genes on the chromosome

Relative positions of genes on the chromosome have also been successfully used to infer functional associations. Most notably, analysis of prokaryotic genomes focused on identifying pairs of orthologs located close to each other on the chromosome, as well as sets of such pairs [10,35,36]. Such clustering is also observed in the eukaryotic genomes, even though they lack well-defined operon structures. A recent study by Lee *et al. *[37] analyzed clustering of genes in KEGG pathways for 5 distant eukaryotic species. The study demonstrated that depending on the genome, 30% to 98% of the pathways exhibit statistically significant levels of gene clustering on the chromosome. A variety of methods have been developed for identifying chromosome gene clusters and evaluating their significance [38]. To generate association scores we use a simple statistical evaluation strategy based on the chromosome gene order, which allows for computationally efficient treatment of large number of genomes (see Methods).

The self-rank performance based on the chromosome clustering association is shown in Figure 4. The overall performance for known *E. coli *metabolic enzymes is better than for the *S. cerevisiae *enzymes, which is expected given the prominent role of operons in prokaryotic transcriptional regulation.

### Other association measures

Interacting proteins encoded by separate genes in some species, may sometimes occur as a single, multi-domain fusion protein in other species. Detecting fusion of non-homologous proteins in another organism has been shown to be a significant predictor of functional association between genes [39-41]. Our calculations of a fusion association score are based on a combination of fusions detected at several sequence homology thresholds (see Methods, Additional file 7. The overall performance of the method is included in Figure 4. Although protein fusion associations are only able to predict relatively small fraction of enzyme-encoding genes (18% for *E. coli*), almost all of predicted enzymes are returned within the top 20 candidates.

A number of metabolic reactions are catalyzed by well established protein complexes, such as the phosphofructokinase complex. Furthermore, metabolic processes commonly involve interactions between multiple metabolic enzymes. For instance, the phosphofructokinase alpha subunit encoded by Pfk1 also interacts with a product of Fba1, fructose-biphosphate adolase II, catalyzing an adjacent reaction in the glycolysis pathway [42]. Large protein-protein interaction datasets have been generated by studies using yeast two-hybrid systems [43,44] and, more recently, mass spectrometry-based techniques [45,46]. In the framework of our approach, candidate genes can be evaluated by assessing the overall amount of interactions between a candidate gene and the metabolic network neighborhood of a missing enzyme. To assess confidence of individual interactions, our analysis makes use of the probabilistic protein interaction dataset from Jansen *et al*. [13], which combines results of four high-throughput interaction datasets [43-46]. The performance of our prediction method on the protein interaction data is significantly lower than that of other association scores, nevertheless it is above of what is expected from a random association score (Figure 4b).

Functional association can be also assessed through similarity of deletion mutant phenotypes under a large set of environmental conditions. For example, deletions of genes that are adjacent to each other in a linear metabolic pathway are likely to result in identical mutant phenotypes. A recent work by Dudley *et al. *[47] experimentally measured growth phenotypes of 4710 *S. cerevisiae *mutants under 21 experimental conditions, including different carbon sources, nutrient limitations, stress and others conditions. We tested the performance of our prediction algorithm on a set of 53 known metabolic enzyme-encoding genes for which high-confidence data was available (see Additional file 8). While the results illustrate predictive power of phenotypic profile associations, overall contribution of this score to the predictions of unidentified enzyme-encoding genes is very small. This is expected, because available high-confidence phenotypic data covers only 14% of *S. cerevisiae *genes.

### Overall enhancements of the individual association scores

Description of a metabolic network neighborhood can be enhanced by considering relative strength of metabolic connections established by different metabolites. Metabolites connecting many enzyme-encoding genes pairs establish, on average, weaker functional associations [20]. The performance of our predictive method can be improved by weighting the contribution of each neighbor in evaluating the overall association of a candidate gene with the metabolic network neighborhood of a missing enzyme. The weight is assigned according to the total number of enzyme pairs associated with a connecting metabolite (see Methods).

Distributions of association scores between a given gene and all other genes in an organism tend to differ from one gene to another. For instance, a gene whose orthologs can be identified in many organisms will typically have more high-confidence chromosome clustering associations than a gene with relatively few detected orthologs. This introduces bias when evaluating overall association with a metabolic network neighborhood. The association-rank rescaling (see Methods) reduces this bias by translating raw association scores into probabilities of metabolic adjacency, calculated based on the rank of raw association score within a distribution of all scores for a particular gene. The rescaling procedure also reduces the number of false positives by considering raw association score of a gene pair with respect to organism-wide score distributions of both genes and choosing a more conservative adjacency probability value.

The effect of metabolite-weighting and association-rank corrections on the self-rank performance is shown in Additional file 9. The predictive performance of all association scores is improved by either correction, with the exception of the protein fusion score, where application of metabolite weighting results in weaker performance.

### Predictions based on combined association evidence

Enzyme-encoding gene predictions based on the individual association scores can be combined to achieve better performance. Normalizing relative strength of different association scores requires informative priors. Such priors can be either constructed manually, for example by consulting experts [12], or learned from known test-cases. This problem has been extensively considered with respect to confidence in pair-wise gene functional associations, and test cases for learning the priors were based on known functional groupings, such as GO annotations [48] or membership in KEGG pathways [10]. For the current problem of prioritizing enzyme-encoding gene candidates, such priors can be learned from known enzyme-encoding genes [6].

Towards the goal of integrating multiple types of association evidence, we have developed two distinct methods. The first approach is based on a *direct likelihood-ratio *(DLR) evaluation of the association score probability distributions. The likelihood that a given candidate gene encodes the desired metabolic enzyme is calculated under the simplifying assumptions that individual association scores are independent and monotonic. The monotonic assumption states that for every association score, the likelihood of association increases monotonically with the absolute value of the score. Both assumptions allow for useful approximations, but in general can be shown to be incorrect. For example, clustering of genes on the chromosomes in *E. coli *is statistically significantly correlated with the similarity in expression profiles (Spearman rank correlation P value < 10^-10^), violating the independence assumption. The DLR method calculates overall likelihood ratio of a candidate gene encoding the desired enzyme as a product of likelihood ratios for each individual association score (see Methods).

The second approach uses a general machine learning method called Adaboost [49,50], and does not rely on independence or monotonicity of the association scores. The generated classifiers are in the form of *alternating decision trees *(ADT), which are generalization of decision stumps, decision trees, and their combination [51] (see Additional file 10). In addition to flexible semantic representation, ADT-based classifiers provide a real-valued measure of confidence, called *classification margin*, which can be related to the probability of a given classification being correct [52]. The Adaboost method has been successfully applied to several large-scale biological problems, including detection of transcription factor binding motifs and prediction of regulatory response [53,54].

**Figure 3:**
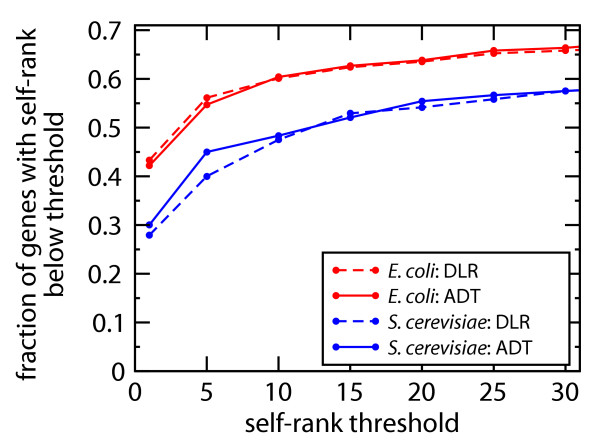
Comparison of ADT and DLR methods for combining multiple association evidence types. Fraction of enzymes predicted within different self-rank thresholds is shown for *E. coli *and *S. cerevisiae *metabolic enzymes. Predictions are based on the combined association evidence (see Methods, Table 1), using two different methods: DLR (dashed curves), and ADT (solid curves).

We find that in identifying missing metabolic genes both ADT and DLR methods achieve comparable levels of performance (Figure 3). The ADT method performs slightly better on *E. coli *metabolic enzymes, and DLR on *S. cerevisiae*. Success of the DLR method relative to a general classifier, such as ADT, suggests that the derived association scores are largely consistent with the underlying assumptions of monotonicity and independence, and allow quality predictions to be made based on a straightforward evaluation of the score probability distributions. The ADT method, however, does not require such assumptions, and may be used to incorporate in the future a wide variety of unrestricted descriptors, such as sequence homology data or expression variability [19].

**Figure 4:**
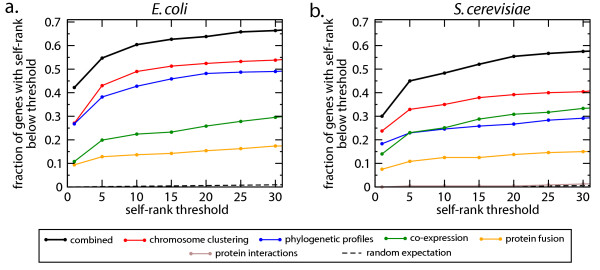
Enzyme predictions based on individual and combined types of association evidence (see Methods, Table 1). Fraction of known enzymes predicted within different self-rank thresholds is shown for **a. ***E. coli *metabolism and **b. ***S. cerevisiae *metabolism. Each curve indicates a probability (y axis) with which a true enzyme-encoding gene will be predicted within top n (x axis) candidates for its enzymatic function. The total number of candidates is 3352 for *E. coli *and 5253 for *S. cerevisiae*. Different curves demonstrate predictive performance of various types of association evidence. Predictions are generated based on functional association with the first three layers of the metabolic network neighborhood, using ADT classifier with 10-fold validation.

Prediction performance of individual functional association scores and their combination using ADT method is shown for *E. coli *metabolic enzymes in Figure 4a. The figure illustrates that predictions based on the combined evidence are clearly superior to what is achieved by any individual type of functional association evidence, with 43% of known enzymes predicted as number one candidates for their enzymatic function, and 60% within the top 10 candidates. Associations based on the chromosome clustering provide the best predictions of any single evidence type, and are able to predict almost half of the metabolic enzymes within the top 10 candidates. It is also important to note that different association evidence types are not redundant – none of the predictions based on a particular association score are completely covered by the predictions of another association score (see Additional file 11). Predictions for 16 unknown and recently identified enzyme-encoding genes that are specified as missing in the *E. coli *metabolic model are given in Additional file 17.

Individual and combined prediction performance for enzymes of *S. cerevisiae *metabolic network is illustrated in Figure 4b. Relative to *E. coli *predictions, co-expression score in *S. cerevisiae *tends to perform better; however chromosome clustering and phylogenetic profile association scores perform worse. The overall level of performance is also lower, with approximately 60% of the enzymes predicted within top 50 candidates (compared to 71% in *E. coli*). The performance difference can be partially attributed to lower number of candidate genes in *E. coli *(3351 as opposed to 5252 in *S. cerevisiae*) and wider availability of the genomic data for bacterial organisms. For instance, chromosome clustering associations were calculated on a dataset that contains nearly a hundred bacterial species and only a handful of eukaryotic genomes.

As earlier studies have utilized pre-defined metabolic pathways to establish functional context of a missing gene, we compared performance of predictions based on layered metabolic neighborhoods with predictions based on KEGG pathway membership. A set of KEGG metabolic pathways for a particular organism provides a list of reactions and enzymes analogous to the *E. coli *and *S. cerevisiae *metabolic models used throughout this manuscript. Such pathways also represent pre-defined, functionally meaningful partitions of the metabolic network. To compare predictive performance, the candidates were evaluated based on the functional associations with genes in the relevant KEGG pathway, instead of the metabolic network neighborhood. We find that associations with layered metabolic neighborhoods are more informative in both *E. coli *and *S. cerevisiae *metabolic models than associations with enzymes in shared KEGG pathways (Additional file 12). For *E. coli *the difference in fraction of predicted enzymes is greatest at low self-ranks (200% at self-rank of 1), and decreases for higher self-ranks (18% at self-rank of 50). This is expected because metabolic neighborhoods are determined specifically for the desired enzymatic function and prioritize neighbors into layers of decreasing functional relevance.

## Conclusion

The results presented in this work demonstrate that the gene encoding a specific metabolic function can be effectively identified from combined functional association with the metabolic network neighborhood of the desired function. This indicates that the relationships established by the local structure of the metabolic network impose constraints on a wide range of natural processes, such as gene expression or evolutionary processes on both molecular and genomic scales. Our tests used a combination of genome context and expression data to identify known *E. coli *metabolic enzymes, predicting them within the top 10 (out of 3352) candidates in 60% of the cases. We show that in the case of both *E. coli *and *S. cerevisiae*, combining multiple types of association evidence results in a significantly better prediction performance than that of any individual association score.

In validating the performance of our method, we relied on the functional associations established by the metabolic network neighborhood as the sole source of information about the desired enzymatic activity. In practice, additional clues regarding activity or physical properties of the unidentified enzyme can be often used to narrow down the set of candidates. These additional clues may provide restrictions on the phylogenetic profile pattern, protein size, presence or absence of membrane spanning regions or specific protein domains. For example, the recently identified *E. coli *arabinose-5-phosphate isomerase, *yrbH *[55], is predicted as a 10^th ^candidate among all genes, but is the only candidate within the top 50 with a putative sugar isomerase domain (see Additional file 17).

The presented approach is limited in its ability to predict additional functions for the enzyme-encoding genes already present in the metabolic model. Specifically, evaluating an enzyme-encoding gene from the metabolic neighborhood of a desired enzymatic function as a candidate for that function, would typically result in a high score regardless of whether the gene actually encodes a desired enzyme.

Sequence homology to known proteins remains the primary method of identifying missing enzymes [4,56]. Predictions based on the association evidence considered in this work are complementary to homology-based methods, and can be used to target enzymes that have not been identified in any organism (referred to as *globally missing enzymes *by Osterman *et al*. [4]). Integration of genome context information into the refined sequence homology searches has been shown to improve the predictions [6]. It will be important to analyze how incorporation of diverse association evidence presented in this work would improve the performance, in particular in with respect to the difficult cases of weak or ambiguous sequence homology. The overall performance of the presented method can be improved in a number of ways. The datasets underlying individual scores can be expanded. Genome divergence corrections for the chromosome clustering score are also likely to improve the results. Further extensions can provide better identification in the cases where multiple missing genes appear within the same metabolic neighborhood. This should be particularly helpful for enzyme identification in poorly studied organisms. In such organisms, the performance of the method will be determined by how much is known about the metabolic neighborhood of the specific enzymatic function. We hope that the presented method, and its future derivations, will be important in completing metabolic models of different organisms.

## Methods

### Metabolic neighborhoods and network representation

Metabolic network was represented as a graph, with nodes corresponding to metabolic enzyme-encoding genes and edges to connections established by the metabolic reactions [20]. Two metabolic genes are connected if the enzymes they encode share a metabolite among the set of reactants or products of the reactions they catalyze. Metabolic *network distance *between enzyme-encoding genes is calculated as a shortest path in the graph. Distance of directly connected genes is taken to be 1. A *metabolic neighborhood layer *of a radius *R *around a metabolic enzyme *X *is defined as a set of all enzyme-encoding genes that are at the distance *R *from the enzyme *X*. A *metabolic neighborhood *of radius *R *is a set of neighborhood layers of radii *r *≤ *R *(Figure 1a).

Detailed metabolic models of *E. coli *[2] and *S. cerevisiae *[21] were used to compile comprehensive connectivity graphs for these organisms, excluding metabolic connections established by the following top 14 most common metabolites: ATP, ADP, AMP, CO_2_, CoA, glutamate, H, NAD, NADH, NADP, NADPH, NH_3_, orthophosphate and pyrophosphate (and corresponding mitochondrial and external species). Common metabolites tend to connect enzymes with weak functional associations [20], and their exclusion generally improves enzyme-encoding gene predictions. The exclusion threshold was chosen to cover majority of common co-factors. We note that overall performance results are not sensitive to the exact set of excluded metabolites (Additional file 13 compares self-rank performance excluding top 7 and top 20 metabolites). However, changes in the metabolite set can affect prediction of individual enzymes, in particular those catalyzing key reactions of the excluded metabolites.

### Self-rank validation

To assess performance of our method we use self-rank measure, which quantifies the ability to predict known metabolic enzymes. A self-rank of a known enzyme-encoding gene is defined as a rank of that gene among a set of candidates in an ordering determined by our algorithm (Figure 1b). A set of candidates consist of all genes in the organism that do not already appear in the metabolic graph (i.e. non-metabolic genes) and the known enzyme-encoding gene that is being tested. Candidate set for *E. coli *contained 3351 open reading frames (ORFs), and for *S. cerevisiae *5252 ORFs. A perfect prediction algorithm would result in a self-rank of 1 (top candidate) for every metabolic enzyme, and a completely non-informative method would result in a uniform distribution of ranks (on the range from 1 to the size of the candidate set).

The overall performance of the method was measured by evaluating self-ranks of a set of known enzyme-encoding genes. The test set includes all enzymes with non-empty metabolic neighborhoods, with the following exceptions: test set excludes enzymes from known multi-subunit complexes, as strong functional association between members of the same complex would lead to overestimation of algorithm performance; test set also excludes enzymes that have high sequence homology (BLASTp E value below 10^-10^) to some other known metabolic enzyme in that organism (paralogs). The exclusion of such paralogous pairs aims to avoid bias stemming from overlapping ortholog mappings. The resulting set contained 351 enzymes from *E. coli *metabolism, and 240 from *S. cerevisiae*.

While paralog filtering allows to minimize bias from overlapping ortholog mappings, it also excludes a significant fraction of known enzymes (50% for *E. coli*, 62% for *S. cerevisiae*), which in itself can be a source of bias. To test this we calculated algorithm performance omitting individual associations between paralogous gene pairs, as an alternative to removing all paralogs from the test set. We find that algorithm performance with and without paralog filtering is comparable for both *E. coli *and *S. cerevisiae *(see Additional file 15).

### Orthology datasets

KEGG ortholog dataset was retrieved from SSDB database (01/2005) [28]. All available closest homologs and best bi-directional hits of *E. coli *and *S. cerevisiae *genes were recorded. BLAST-based dataset was constructed using BLASTp queries against NCBI NR protein dataset (03/2005), using E-value cutoff of 10^-3 ^and limiting the maximum number of homologs per query to 6000. To determine best bi-directional hits, reverse BLASTp queries were run for every hit against target genome (*E. coli *or *S. cerevisiae*). NCBI taxonomy identifiers were used to group hits belonging to the same organism. For *E. coli *only organisms containing orthologs to more than 4% of genes were considered (7% for *S. cerevisiae*). We found that performance of analogous datasets constructed using TBLASTN queries was similar.

### Phylogenetic profile co-occurrence

Given a set of genomes *G *= {*G*_1_..GNG
 MathType@MTEF@5@5@+=feaafiart1ev1aaatCvAUfKttLearuWrP9MDH5MBPbIqV92AaeXatLxBI9gBaebbnrfifHhDYfgasaacH8akY=wiFfYdH8Gipec8Eeeu0xXdbba9frFj0=OqFfea0dXdd9vqai=hGuQ8kuc9pgc9s8qqaq=dirpe0xb9q8qiLsFr0=vr0=vr0dc8meaabaqaciaacaGaaeqabaqabeGadaaakeaacqWGhbWrdaWgaaWcbaGaemOta40aaSbaaWqaaiabdEeahbqabaaaleqaaaaa@3063@}, a phylogenetic profile of a gene was represented as a binary vector *ε *of length *N*_*G*_, such that *ε*_*i *_= 1 if an orthologous gene is present in genome *G*_*i*_, and *ε*_*i *_= 0 otherwise.

Assuming that orthologs are independently identically distributed (IID) *within *each genome *G*_i_, the probability of observing two profiles of a given similarity under the null hypothesis is calculated using hypergeometric distribution [25]:

P(k|n,m,N)=(nk)(N−nm−k)(Nm)     Equation1
 MathType@MTEF@5@5@+=feaafiart1ev1aaatCvAUfKttLearuWrP9MDH5MBPbIqV92AaeXatLxBI9gBaebbnrfifHhDYfgasaacH8akY=wiFfYdH8Gipec8Eeeu0xXdbba9frFj0=OqFfea0dXdd9vqai=hGuQ8kuc9pgc9s8qqaq=dirpe0xb9q8qiLsFr0=vr0=vr0dc8meaabaqaciaacaGaaeqabaqabeGadaaakeaacqWGqbaucqGGOaakcqWGRbWAcqGG8baFcqWGUbGBcqGGSaalcqWGTbqBcqGGSaalcqWGobGtcqGGPaqkcqGH9aqpdaWcaaqaamaabmaabaqbaeqabiqaaaqaaiabd6gaUbqaaiabdUgaRbaaaiaawIcacaGLPaaadaqadaqaauaabeqaceaaaeaacqWGobGtcqGHsislcqWGUbGBaeaacqWGTbqBcqGHsislcqWGRbWAaaaacaGLOaGaayzkaaaabaWaaeWaaeaafaqabeGabaaabaGaemOta4eabaGaemyBa0gaaaGaayjkaiaawMcaaaaacaWLjaGaaCzcaGqabiab=veafjab=fhaXjab=vha1jab=fgaHjab=rha0jab=LgaPjab=9gaVjab=5gaUjab=bcaGiab=fdaXaaa@580B@

where *k *is the number of ortholog co-occurrences, *N *is the size of the genome set *G*, *n *and *m *correspond to the number of orthologs in the two profiles being compared. The probability of functional association is then given by Passociation=1−∑k>KP(k|n,m,N)
 MathType@MTEF@5@5@+=feaafiart1ev1aaatCvAUfKttLearuWrP9MDH5MBPbIqV92AaeXatLxBI9gBaebbnrfifHhDYfgasaacH8akY=wiFfYdH8Gipec8Eeeu0xXdbba9frFj0=OqFfea0dXdd9vqai=hGuQ8kuc9pgc9s8qqaq=dirpe0xb9q8qiLsFr0=vr0=vr0dc8meaabaqaciaacaGaaeqabaqabeGadaaakeaacqWGqbaudaWgaaWcbaGaemyyaeMaem4CamNaem4CamNaem4Ba8Maem4yamMaemyAaKMaemyyaeMaemiDaqNaemyAaKMaem4Ba8MaemOBa4gabeaakiabg2da9iabigdaXiabgkHiTmaaqafabaGaemiuaa1aaeWaaeaacqWGRbWAcqGG8baFcqWGUbGBcqGGSaalcqWGTbqBcqGGSaalcqWGobGtaiaawIcacaGLPaaaaSqaaiabdUgaRjabg6da+iabdUealbqab0GaeyyeIuoaaaa@50F2@, where *K *is the number of actual ortholog co-occurrences observed between two specific profiles [11].

If the assumption of identical ortholog distribution within each genome is relaxed, probability *P*(*k*|*n*, *m*, *N*) is distributed as a sum of independent, non-identical Bernoulli variables *x*_*i*_: k~∑min⁡(n,m)xi
 MathType@MTEF@5@5@+=feaafiart1ev1aaatCvAUfKttLearuWrP9MDH5MBPbIqV92AaeXatLxBI9gBaebbnrfifHhDYfgasaacH8akY=wiFfYdH8Gipec8Eeeu0xXdbba9frFj0=OqFfea0dXdd9vqai=hGuQ8kuc9pgc9s8qqaq=dirpe0xb9q8qiLsFr0=vr0=vr0dc8meaabaqaciaacaGaaeqabaqabeGadaaakeaacqWGRbWAcqGG+bGFdaaeqbqaaiabdIha4naaBaaaleaacqWGPbqAaeqaaaqaaiGbc2gaTjabcMgaPjabc6gaUjabcIcaOiabd6gaUjabcYcaSiabd2gaTjabcMcaPaqab0GaeyyeIuoaaaa@3E23@, with *p*(*x*_*i*_) corresponding to the probability of observing a match in a given genome *i*. This is a special case of the Extended Multivariable Hypergeometric distribution [26].

Given a gene *x *with ortholog occurrence profile *ε*^*x*^, the probability of observing *k *ortholog co-occurrences between gene *x *and some other gene *y*, *P*(*k*|*n*, *m*, *N*), is calculated using the following recursive approach. Let *P*_*i*_(*k'*|*m'*) be a probability of observing a total of *k' *ortholog co-occurrences between gene *x *and some other gene *y *in the genomes *G*_*j *_such that *j *≤ *i *(*G*_*j*:*j*≤*i*_), where *m' *is the number of orthologs of gene *y *in the genomes *G*_*j*:*j*≤*i*_. Then, by definition, *P*(*k*|*n*, *m*, *N*) = *P*_*N*_(*k*|*m*).

Let pi,m′o
 MathType@MTEF@5@5@+=feaafiart1ev1aaatCvAUfKttLearuWrP9MDH5MBPbIqV92AaeXatLxBI9gBaebbnrfifHhDYfgasaacH8akY=wiFfYdH8Gipec8Eeeu0xXdbba9frFj0=OqFfea0dXdd9vqai=hGuQ8kuc9pgc9s8qqaq=dirpe0xb9q8qiLsFr0=vr0=vr0dc8meaabaqaciaacaGaaeqabaqabeGadaaakeaacqWGWbaCdaqhaaWcbaGaemyAaKMaeiilaWIafmyBa0MbauaaaeaacqWGVbWBaaaaaa@3353@ be a probability of one of the remaining *m' *orthologs of gene *y *occurring in genome *G*_*i*_. Then *P*_*i*_(*k'*|*m'*) can be calculated recursively by considering separately the cases when an ortholog of gene *y *does or does not occur in the genome *G*_*i*_:

*P*_*i*_(*k'*|*m'*) = pi,m′o
 MathType@MTEF@5@5@+=feaafiart1ev1aaatCvAUfKttLearuWrP9MDH5MBPbIqV92AaeXatLxBI9gBaebbnrfifHhDYfgasaacH8akY=wiFfYdH8Gipec8Eeeu0xXdbba9frFj0=OqFfea0dXdd9vqai=hGuQ8kuc9pgc9s8qqaq=dirpe0xb9q8qiLsFr0=vr0=vr0dc8meaabaqaciaacaGaaeqabaqabeGadaaakeaacqWGWbaCdaqhaaWcbaGaemyAaKMaeiilaWIafmyBa0MbauaaaeaacqWGVbWBaaaaaa@3353@*P*_*i*_(*k'*|*m'*, ortholog of *y *occurs in *G*_*i*_) + (1 - pi,m′o
 MathType@MTEF@5@5@+=feaafiart1ev1aaatCvAUfKttLearuWrP9MDH5MBPbIqV92AaeXatLxBI9gBaebbnrfifHhDYfgasaacH8akY=wiFfYdH8Gipec8Eeeu0xXdbba9frFj0=OqFfea0dXdd9vqai=hGuQ8kuc9pgc9s8qqaq=dirpe0xb9q8qiLsFr0=vr0=vr0dc8meaabaqaciaacaGaaeqabaqabeGadaaakeaacqWGWbaCdaqhaaWcbaGaemyAaKMaeiilaWIafmyBa0MbauaaaeaacqWGVbWBaaaaaa@3353@)*P*_*i*_(*k'*|*m'*, ortholog of *y *does not occur in *G*_*i*_)     **Equation 2**

Overall recursive definition of *P*_*i*_(*k'*|*m'*), including base cases is given by Equation 3 below:

{Pi(k′|m′)=pi,m′o[εixPi−1(k′−1|m′−1)+(1−εix)Pi−1(k′|m′−1)]+(1−pi,m′o)Pi−1(k′|m′)P1(1|1)=ε1xPi(0|0)=1Pi(k|m)=0k<0Pi(k|m)=0k>m     Equation3
 MathType@MTEF@5@5@+=feaafiart1ev1aaatCvAUfKttLearuWrP9MDH5MBPbIqV92AaeXatLxBI9gBaebbnrfifHhDYfgasaacH8akY=wiFfYdH8Gipec8Eeeu0xXdbba9frFj0=OqFfea0dXdd9vqai=hGuQ8kuc9pgc9s8qqaq=dirpe0xb9q8qiLsFr0=vr0=vr0dc8meaabaqaciaacaGaaeqabaqabeGadaaakeaadaGabaqaauaabaqafiaaaaqaaiabdcfaqnaaBaaaleaacqWGPbqAaeqaaOWaaeWaaeaacuWGRbWAgaqbaiabcYha8jqbd2gaTzaafaaacaGLOaGaayzkaaGaeyypa0JaemiCaa3aa0baaSqaaiabdMgaPjabcYcaSiqbd2gaTzaafaaabaGaem4Ba8gaaOWaamWaaeaaiiGacqWF1oqzdaqhaaWcbaGaemyAaKgabaGaemiEaGhaaOGaemiuaa1aaSbaaSqaaiabdMgaPjabgkHiTiabigdaXaqabaGcdaqadaqaaiqbdUgaRzaafaGaeyOeI0IaeGymaeJaeiiFaWNafmyBa0MbauaacqGHsislcqaIXaqmaiaawIcacaGLPaaacqGHRaWkdaqadaqaaiabigdaXiabgkHiTiab=v7aLnaaDaaaleaacqWGPbqAaeaacqWG4baEaaaakiaawIcacaGLPaaacqWGqbaudaWgaaWcbaGaemyAaKMaeyOeI0IaeGymaedabeaakmaabmaabaGafm4AaSMbauaacqGG8baFcuWGTbqBgaqbaiabgkHiTiabigdaXaGaayjkaiaawMcaaaGaay5waiaaw2faaiabgUcaRmaabmaabaGaeGymaeJaeyOeI0IaemiCaa3aa0baaSqaaiabdMgaPjabcYcaSiqbd2gaTzaafaaabaGaem4Ba8gaaaGccaGLOaGaayzkaaGaemiuaa1aaSbaaSqaaiabdMgaPjabgkHiTiabigdaXaqabaGcdaqadaqaaiqbdUgaRzaafaGaeiiFaWNafmyBa0MbauaaaiaawIcacaGLPaaaaeaaaeaacqWGqbaudaWgaaWcbaGaeGymaedabeaakmaabmaabaGaeGymaeJaeiiFaWNaeGymaedacaGLOaGaayzkaaGaeyypa0Jae8xTdu2aa0baaSqaaiabigdaXaqaaiabdIha4baaaOqaaaqaaiabdcfaqnaaBaaaleaacqWGPbqAaeqaaOWaaeWaaeaacqaIWaamcqGG8baFcqaIWaamaiaawIcacaGLPaaacqGH9aqpcqaIXaqmaeaaaeaacqWGqbaudaWgaaWcbaGaemyAaKgabeaakmaabmaabaGaem4AaSMaeiiFaWNaemyBa0gacaGLOaGaayzkaaGaeyypa0JaeGimaadabaGaem4AaSMaeyipaWJaeGimaadabaGaemiuaa1aaSbaaSqaaiabdMgaPbqabaGcdaqadaqaaiabdUgaRjabcYha8jabd2gaTbGaayjkaiaawMcaaiabg2da9iabicdaWaqaaiabdUgaRjabg6da+iabd2gaTbaaaiaawUhaaiaaxMaacaWLjaacbeGae4xrauKae4xCaeNae4xDauNae4xyaeMae4hDaqNae4xAaKMae43Ba8Mae4NBa4Mae4hiaaIae43mamdaaa@BD9A@

where εix
 MathType@MTEF@5@5@+=feaafiart1ev1aaatCvAUfKttLearuWrP9MDH5MBPbIqV92AaeXatLxBI9gBaebbnrfifHhDYfgasaacH8akY=wiFfYdH8Gipec8Eeeu0xXdbba9frFj0=OqFfea0dXdd9vqai=hGuQ8kuc9pgc9s8qqaq=dirpe0xb9q8qiLsFr0=vr0=vr0dc8meaabaqaciaacaGaaeqabaqabeGadaaakeaacqaH1oqzdaqhaaWcbaGaemyAaKgabaGaemiEaGhaaaaa@3154@ is the value of the ortholog occurrence profile (0 or 1) of gene *x *in genome *G*_*i*_.

*P*_*i*_(*k'*|*n'*, *m'*) is computed using a dynamic programming approach. The consideration of non-identical distribution of ortholog frequency within each genome is then localized to pi,m′o
 MathType@MTEF@5@5@+=feaafiart1ev1aaatCvAUfKttLearuWrP9MDH5MBPbIqV92AaeXatLxBI9gBaebbnrfifHhDYfgasaacH8akY=wiFfYdH8Gipec8Eeeu0xXdbba9frFj0=OqFfea0dXdd9vqai=hGuQ8kuc9pgc9s8qqaq=dirpe0xb9q8qiLsFr0=vr0=vr0dc8meaabaqaciaacaGaaeqabaqabeGadaaakeaacqWGWbaCdaqhaaWcbaGaemyAaKMaeiilaWIafmyBa0MbauaaaeaacqWGVbWBaaaaaa@3353@, which in this case is distributed according to the marginal Extended Hypergeometric distribution. The marginal form of the distribution is more amenable to the computational approximations than the regular form. Since pi,m′o
 MathType@MTEF@5@5@+=feaafiart1ev1aaatCvAUfKttLearuWrP9MDH5MBPbIqV92AaeXatLxBI9gBaebbnrfifHhDYfgasaacH8akY=wiFfYdH8Gipec8Eeeu0xXdbba9frFj0=OqFfea0dXdd9vqai=hGuQ8kuc9pgc9s8qqaq=dirpe0xb9q8qiLsFr0=vr0=vr0dc8meaabaqaciaacaGaaeqabaqabeGadaaakeaacqWGWbaCdaqhaaWcbaGaemyAaKMaeiilaWIafmyBa0MbauaaaeaacqWGVbWBaaaaaa@3353@ does not depend on the choice of genes *x *and *y*, we sample pi,m′o
 MathType@MTEF@5@5@+=feaafiart1ev1aaatCvAUfKttLearuWrP9MDH5MBPbIqV92AaeXatLxBI9gBaebbnrfifHhDYfgasaacH8akY=wiFfYdH8Gipec8Eeeu0xXdbba9frFj0=OqFfea0dXdd9vqai=hGuQ8kuc9pgc9s8qqaq=dirpe0xb9q8qiLsFr0=vr0=vr0dc8meaabaqaciaacaGaaeqabaqabeGadaaakeaacqWGWbaCdaqhaaWcbaGaemyAaKMaeiilaWIafmyBa0MbauaaaeaacqWGVbWBaaaaaa@3353@ computationally, taking into account individual ortholog occurrence frequencies of each genome. The probability of ortholog occurrence in a specific genome (pi,m′o
 MathType@MTEF@5@5@+=feaafiart1ev1aaatCvAUfKttLearuWrP9MDH5MBPbIqV92AaeXatLxBI9gBaebbnrfifHhDYfgasaacH8akY=wiFfYdH8Gipec8Eeeu0xXdbba9frFj0=OqFfea0dXdd9vqai=hGuQ8kuc9pgc9s8qqaq=dirpe0xb9q8qiLsFr0=vr0=vr0dc8meaabaqaciaacaGaaeqabaqabeGadaaakeaacqWGWbaCdaqhaaWcbaGaemyAaKMaeiilaWIafmyBa0MbauaaaeaacqWGVbWBaaaaaa@3353@) was sampled computationally by drawing from the set of organisms without replacement with relative probabilities corresponding to the rate of ortholog occurrences in each genome. In each iteration draws were performed until all of the organisms were drawn. A total of 10^6 ^such iterations were performed. The sensitivity was assessed using *E. coli *phylogenetic profile data (BLAST-based dataset). At 10^6 ^iterations, the mean standard error of pi,m′o
 MathType@MTEF@5@5@+=feaafiart1ev1aaatCvAUfKttLearuWrP9MDH5MBPbIqV92AaeXatLxBI9gBaebbnrfifHhDYfgasaacH8akY=wiFfYdH8Gipec8Eeeu0xXdbba9frFj0=OqFfea0dXdd9vqai=hGuQ8kuc9pgc9s8qqaq=dirpe0xb9q8qiLsFr0=vr0=vr0dc8meaabaqaciaacaGaaeqabaqabeGadaaakeaacqWGWbaCdaqhaaWcbaGaemyAaKMaeiilaWIafmyBa0MbauaaaeaacqWGVbWBaaaaaa@3353@ is 3.0·10^-4 ^(estimated from 100 independent runs). The mean standard error of the resulting self-ranks of known enzyme-encoding genes is 0.70, and standard errors for the fraction of known enzyme-encoding genes predicted below self-rank thresholds of 10, 20 and 50 is < 10^-10^, 8.5·10^-4^and 1.4·10^-3 ^respectively.

To correct for non-independent ortholog occurrence rates, we first evaluate the distance between a pair of query genomes *X *and *Y *as:

d(X,Y)=MI(X,Y)min⁡(H(X),H(Y))     Equation4
 MathType@MTEF@5@5@+=feaafiart1ev1aaatCvAUfKttLearuWrP9MDH5MBPbIqV92AaeXatLxBI9gBaebbnrfifHhDYfgasaacH8akY=wiFfYdH8Gipec8Eeeu0xXdbba9frFj0=OqFfea0dXdd9vqai=hGuQ8kuc9pgc9s8qqaq=dirpe0xb9q8qiLsFr0=vr0=vr0dc8meaabaqaciaacaGaaeqabaqabeGadaaakeaacqWGKbazdaqadaqaaiabdIfayjabcYcaSiabdMfazbGaayjkaiaawMcaaiabg2da9maalaaabaGaemyta0KaemysaK0aaeWaaeaacqWGybawcqGGSaalcqWGzbqwaiaawIcacaGLPaaaaeaacyGGTbqBcqGGPbqAcqGGUbGBdaqadaqaaiabdIeainaabmaabaGaemiwaGfacaGLOaGaayzkaaGaeiilaWIaemisaG0aaeWaaeaacqWGzbqwaiaawIcacaGLPaaaaiaawIcacaGLPaaaaaGaaCzcaiaaxMaaieqacqWFfbqrcqWFXbqCcqWF1bqDcqWFHbqycqWF0baDcqWFPbqAcqWFVbWBcqWFUbGBcqWFGaaicqWF0aanaaa@5702@

where *MI*(*X*, *Y*) is mutual information between ortholog occurrence vectors for genomes *X *and *Y*, and *H*() is Shannon entropy of each vector. The ortholog occurrence vector for a query genome *X *is a binary vector of length *N*_*genes *_(number of genes in a target organism, i.e. *E. coli*), such that value of the *i*^th ^element is 1 if ortholog of an *i*^th ^gene is found in *X*, and 0 otherwise. Clusters of closely related organisms (*d*(*X*, *Y*) < 0.8, Equation 4) were determined by neighbor-joining method [57]. Several ways of summarizing the ortholog co-occurrence vector for a cluster of closely related organisms were tested: selecting organism with highest entropy, using AND/OR functions, and using majority rule. We find that performance of AND function is optimal for the threshold of *d*(*X*, *Y*) < 0.8, however for higher thresholds selecting an organism with highest entropy results in better performance.

In evaluating performance without adjacency-rank rescaling (i.e. Figure 2), total phylogenetic profile association score between a candidate gene *x *and a metabolic neighborhood layer *L *was calculated as:

scoreL(x)=∑g∈L1P(x,g)     Equation5
 MathType@MTEF@5@5@+=feaafiart1ev1aaatCvAUfKttLearuWrP9MDH5MBPbIqV92AaeXatLxBI9gBaebbnrfifHhDYfgasaacH8akY=wiFfYdH8Gipec8Eeeu0xXdbba9frFj0=OqFfea0dXdd9vqai=hGuQ8kuc9pgc9s8qqaq=dirpe0xb9q8qiLsFr0=vr0=vr0dc8meaabaqaciaacaGaaeqabaqabeGadaaakeaacqWGZbWCcqWGJbWycqWGVbWBcqWGYbGCcqWGLbqzdaWgaaWcbaGaemitaWeabeaakmaabmaabaGaemiEaGhacaGLOaGaayzkaaGaeyypa0ZaaabuaeaadaWcaaqaaiabigdaXaqaaiabdcfaqnaabmaabaGaemiEaGNaeiilaWIaem4zaCgacaGLOaGaayzkaaaaaaWcbaGaem4zaCMaeyicI4SaemitaWeabeqdcqGHris5aOGaaCzcaiaaxMaaieqacqWFfbqrcqWFXbqCcqWF1bqDcqWFHbqycqWF0baDcqWFPbqAcqWFVbWBcqWFUbGBcqWFGaaicqWF1aqnaaa@5431@

where *P*(*x*, *g*) is the probability of observing a given number of ortholog co-occurrences between genes *x *and *g*, calculated using hypergeometric or extended hypergeometric distribution. Functional form given in Equation 5 will assign high scores to candidates with strong functional associations (i.e. very low values of *P*(*x*, *g*)) to the genes in the metabolic network neighborhood layer *L*. Other functional forms, including those with optimized parameters can be used ([19], Chen and Vitkup, submitted).

To estimate self-rank performance of the COG dataset correcting for the bias in orthology dataset coverage (Figure 2b), the fraction of true enzyme-encoding genes, *f *predicted within a particular self-rank threshold *t *was calculated as *f*(*t*) = *α*_*M *_*f'*(*α*_*C*_*t*), where *α*_*M *_is the fraction of test metabolic enzyme-encoding genes covered by the COG dataset, *α*_*C *_is the fraction of candidate set genes (non-metabolic) covered by the dataset, and *f' *is the performance on the set of metabolic and candidate genes covered by the COG dataset.

### Gene co-expression

Co-expression association value was calculated as Spearman rank correlation [58] between expression profiles. *E. coli *co-expression was calculated based on the 180 conditions from the Stanford Microarray Database (SMD dataset) [59]. *S. cerevisiae *co-expression was measured based on the mRNA expression profiles from Rosetta "compendium" dataset [60]. Log10 intensity ratio data was used. Co-expression of orthologous genes was determined using KEGG ortholog dataset.

### Clustering on the chromosome

The degree to which orthologs of two genes are clustered on the chromosome was calculated based on the null hypothesis that genes are randomly distributed across the chromosomes. Instead of considering gene sizes and exact nucleotide positions, we concentrated on gene order statistics.

Given a pair of genes *x *and *y*, we define *P*(*d*_*g*_(*x*, *y*)) as the probability of observing gene order distance *d*_*g*_(*x*, *y*) or smaller between orthologs of genes *x *and *y *in a genome *g*. Under the null hypothesis, *P*(*d*_g_(*x*, *y*)) is calculated directly, by counting the number of gene pairs in the organism *g *that are separated by gene order distance *d*_g_(*x*, *y*) or smaller (taking into account chromosome sizes in *g*). Clustering of genes *x *and *y *in a set of query genomes *G *was calculated as PG(x,y)=∏g∈GP(dg(x,y))
 MathType@MTEF@5@5@+=feaafiart1ev1aaatCvAUfKttLearuWrP9MDH5MBPbIqV92AaeXatLxBI9gBaebbnrfifHhDYfgasaacH8akY=wiFfYdH8Gipec8Eeeu0xXdbba9frFj0=OqFfea0dXdd9vqai=hGuQ8kuc9pgc9s8qqaq=dirpe0xb9q8qiLsFr0=vr0=vr0dc8meaabaqaciaacaGaaeqabaqabeGadaaakeaacqWGqbaudaWgaaWcbaGaem4raCeabeaakmaabmaabaGaemiEaGNaeiilaWIaemyEaKhacaGLOaGaayzkaaGaeyypa0ZaaebuaeaacqWGqbaudaqadaqaaiabdsgaKnaaBaaaleaacqWGNbWzaeqaaOWaaeWaaeaacqWG4baEcqGGSaalcqWG5bqEaiaawIcacaGLPaaaaiaawIcacaGLPaaaaSqaaiabdEgaNjabgIGiolabdEeahbqab0Gaey4dIunaaaa@4676@. The association strength between of a candidate gene *x *for a metabolic neighborhood layer *N*_*l *_was calculated as P(x|Nl)=∏y∈NlPG(x,y)
 MathType@MTEF@5@5@+=feaafiart1ev1aaatCvAUfKttLearuWrP9MDH5MBPbIqV92AaeXatLxBI9gBaebbnrfifHhDYfgasaacH8akY=wiFfYdH8Gipec8Eeeu0xXdbba9frFj0=OqFfea0dXdd9vqai=hGuQ8kuc9pgc9s8qqaq=dirpe0xb9q8qiLsFr0=vr0=vr0dc8meaabaqaciaacaGaaeqabaqabeGadaaakeaacqWGqbaudaqadaqaaiabdIha4jabcYha8jabd6eaonaaBaaaleaacqWGSbaBaeqaaaGccaGLOaGaayzkaaGaeyypa0ZaaebuaeaacqWGqbaudaWgaaWcbaGaem4raCeabeaakmaabmaabaGaemiEaGNaeiilaWIaemyEaKhacaGLOaGaayzkaaaaleaacqWG5bqEcqGHiiIZcqWGobGtdaWgaaadbaGaemiBaWgabeaaaSqab0Gaey4dIunaaaa@45BB@. The above formulation is based on two major assumptions: (1) gene order distances to different genes of the neighborhood layer *N*_*l *_are independent, and (2) gene order distances between a specific pair of genes are independent across different organisms.

Given a large number of genomes, this evaluation measure will be biased by the variable divergence of different organisms from each other. For example, a set of genes whose orthologs appear only in several closely-related organisms will appear to be more clustered than the set of genes spanning more distant genomes. General probabilistic correction for the genome divergence depends on the details of the rearrangement regime, and presents considerable computational difficulties [38,61]. To minimize the effect of variable genome divergence we have removed from consideration genomes of closely related species. We also note that the bias in the prediction performance is likely to be minimal, as addition of 5 closely related species did not affect the results (data not shown).

The results are based on a set of 105 bacterial and three eukaryotic genomes (*S. cerevisiae*, *S. pombe*, *C. elegans*) from Genbank. The set was screened to eliminate closely related species using ortholog occurrence mutual information threshold of 0.9. Orthology mapping was established using KEGG-based dataset, with best bi-directional hits.

We note that evaluation of chromosome clustering based on the nucleotide positions (as opposed to gene order) produces comparable results (see Additional file 14).

### Protein interactions

Interaction likelihood ratios from the PIE dataset by Jansen *et al*. [13] were used as pair-wise protein interaction association values.

### Protein fusions

Two proteins *x *and *y *of a target genome (*S. cerevisiae *and *E. coli*) were taken to be associated through a protein fusion event if both of the following conditions were met:

1.) *x *and *y *are homologous to the same protein *z *in one of the query genomes with a BLASTp E value below a specified threshold (*E*_*threshold*_), and with at least 70% of their sequences aligned to *z*.

2.) *x *and *y *align to different regions of *z*, or to regions overlapped by no more than 10% of the shorter protein among *x *and *y*. If *x *or *y *align to multiple regions of *z*, then any two regions must not overlap.

A set of 70 query genomes, based on the study by Bowers *et al*. [11], was downloaded from the Entrez Genome database[62]. Several values of *E*_*threshold *_were used in generating enzyme-encoding gene predictions (Figures 3 and 4), with *E*_*threshold *_= 10^-2^, 10^-5 ^and 10^-10 ^for *E. coli*; *E*_*threshold *_= 10^-3^, 10^-5 ^and 10^-10 ^for *S. cerevisiae*.

### Adjacency-rank score rescaling, metabolite weighting and calculation of layer association scores

To perform adjacency-rank score rescaling of raw pair-wise association values, we calculate likelihood ratio of metabolic adjacency (*alr*) for a pair of genes *x *and *y*:

alr(x,y)=Ngenesmax⁡{rxy,ryx}Padj(r≤max⁡{rxy,ryx})     Equation6
 MathType@MTEF@5@5@+=feaafiart1ev1aaatCvAUfKttLearuWrP9MDH5MBPbIqV92AaeXatLxBI9gBaebbnrfifHhDYfgasaacH8akY=wiFfYdH8Gipec8Eeeu0xXdbba9frFj0=OqFfea0dXdd9vqai=hGuQ8kuc9pgc9s8qqaq=dirpe0xb9q8qiLsFr0=vr0=vr0dc8meaabaqaciaacaGaaeqabaqabeGadaaakeaacqWGHbqycqWGSbaBcqWGYbGCdaqadaqaaiabdIha4jabcYcaSiabdMha5bGaayjkaiaawMcaaiabg2da9maalaaabaGaemOta40aaSbaaSqaaiabdEgaNjabdwgaLjabd6gaUjabdwgaLjabdohaZbqabaaakeaacyGGTbqBcqGGHbqycqGG4baEdaGadaqaaiabdkhaYnaaDaaaleaacqWG4baEaeaacqWG5bqEaaGccqGGSaalcqWGYbGCdaqhaaWcbaGaemyEaKhabaGaemiEaGhaaaGccaGL7bGaayzFaaaaaiabdcfaqnaaBaaaleaacqWGHbqycqWGKbazcqWGQbGAaeqaaOWaaeWaaeaacqWGYbGCcqGHKjYOcyGGTbqBcqGGHbqycqGG4baEdaGadaqaaiabdkhaYnaaDaaaleaacqWG4baEaeaacqWG5bqEaaGccqGGSaalcqWGYbGCdaqhaaWcbaGaemyEaKhabaGaemiEaGhaaaGccaGL7bGaayzFaaaacaGLOaGaayzkaaGaaCzcaiaaxMaaieqacqWFfbqrcqWFXbqCcqWF1bqDcqWFHbqycqWF0baDcqWFPbqAcqWFVbWBcqWFUbGBcqWFGaaicqWF2aGnaaa@77ED@

where ryx
 MathType@MTEF@5@5@+=feaafiart1ev1aaatCvAUfKttLearuWrP9MDH5MBPbIqV92AaeXatLxBI9gBaebbnrfifHhDYfgasaacH8akY=wiFfYdH8Gipec8Eeeu0xXdbba9frFj0=OqFfea0dXdd9vqai=hGuQ8kuc9pgc9s8qqaq=dirpe0xb9q8qiLsFr0=vr0=vr0dc8meaabaqaciaacaGaaeqabaqabeGadaaakeaacqWGYbGCdaqhaaWcbaGaemyEaKhabaGaemiEaGhaaaaa@313A@ is a rank of gene *y *among a set of raw association values between gene *x *and all other genes in the organism. Lower ranks correspond to higher stringency of association. *N*_*genes *_is the number of genes in an organism. The probability Padj(r≤max⁡{rxy,ryx})
 MathType@MTEF@5@5@+=feaafiart1ev1aaatCvAUfKttLearuWrP9MDH5MBPbIqV92AaeXatLxBI9gBaebbnrfifHhDYfgasaacH8akY=wiFfYdH8Gipec8Eeeu0xXdbba9frFj0=OqFfea0dXdd9vqai=hGuQ8kuc9pgc9s8qqaq=dirpe0xb9q8qiLsFr0=vr0=vr0dc8meaabaqaciaacaGaaeqabaqabeGadaaakeaacqWGqbaudaWgaaWcbaGaemyyaeMaemizaqMaemOAaOgabeaakmaabmaabaGaemOCaiNaeyizImQagiyBa0MaeiyyaeMaeiiEaG3aaiWaaeaacqWGYbGCdaqhaaWcbaGaemiEaGhabaGaemyEaKhaaOGaeiilaWIaemOCai3aa0baaSqaaiabdMha5bqaaiabdIha4baaaOGaay5Eaiaaw2haaaGaayjkaiaawMcaaaaa@4716@ is calculated as a fraction of metabolically adjacent (i.e. directly connected) gene pairs with association rank below max max⁡{rxy,ryx}
 MathType@MTEF@5@5@+=feaafiart1ev1aaatCvAUfKttLearuWrP9MDH5MBPbIqV92AaeXatLxBI9gBaebbnrfifHhDYfgasaacH8akY=wiFfYdH8Gipec8Eeeu0xXdbba9frFj0=OqFfea0dXdd9vqai=hGuQ8kuc9pgc9s8qqaq=dirpe0xb9q8qiLsFr0=vr0=vr0dc8meaabaqaciaacaGaaeqabaqabeGadaaakeaacyGGTbqBcqGGHbqycqGG4baEdaGadaqaaiabdkhaYnaaDaaaleaacqWG4baEaeaacqWG5bqEaaGccqGGSaalcqWGYbGCdaqhaaWcbaGaemyEaKhabaGaemiEaGhaaaGccaGL7bGaayzFaaaaaa@3D13@ :

Padj(r≤R)=|rba≤R|(a,b)∈A|A|     Equation7
 MathType@MTEF@5@5@+=feaafiart1ev1aaatCvAUfKttLearuWrP9MDH5MBPbIqV92AaeXatLxBI9gBaebbnrfifHhDYfgasaacH8akY=wiFfYdH8Gipec8Eeeu0xXdbba9frFj0=OqFfea0dXdd9vqai=hGuQ8kuc9pgc9s8qqaq=dirpe0xb9q8qiLsFr0=vr0=vr0dc8meaabaqaciaacaGaaeqabaqabeGadaaakeaacqWGqbaudaWgaaWcbaGaemyyaeMaemizaqMaemOAaOgabeaakmaabmaabaGaemOCaiNaeyizImQaemOuaifacaGLOaGaayzkaaGaeyypa0ZaaSaaaeaadaabdaqaaiabdkhaYnaaDaaaleaacqWGIbGyaeaacqWGHbqyaaGccqGHKjYOcqWGsbGuaiaawEa7caGLiWoadaWgaaWcbaWaaeWaaeaacqWGHbqycqGGSaalcqWGIbGyaiaawIcacaGLPaaacqGHiiIZcqWGbbqqaeqaaaGcbaWaaqWaaeaacqWGbbqqaiaawEa7caGLiWoaaaGaaCzcaiaaxMaaieqacqWFfbqrcqWFXbqCcqWF1bqDcqWFHbqycqWF0baDcqWFPbqAcqWFVbWBcqWFUbGBcqWFGaaicqWF3aWnaaa@5CDF@

where A is a set of all gene pairs (*a*, *b*) such that *a *and *b *are directly connected in the metabolic graph, excluding pairs involving *x *or *y*.

Without metabolite weighting, the total association score between a candidate gene *x *and a metabolic neighborhood layer *L *is calculated as scoreL(x)=∑g∈Lexp⁡[alr(x,g)]
 MathType@MTEF@5@5@+=feaafiart1ev1aaatCvAUfKttLearuWrP9MDH5MBPbIqV92AaeXatLxBI9gBaebbnrfifHhDYfgasaacH8akY=wiFfYdH8Gipec8Eeeu0xXdbba9frFj0=OqFfea0dXdd9vqai=hGuQ8kuc9pgc9s8qqaq=dirpe0xb9q8qiLsFr0=vr0=vr0dc8meaabaqaciaacaGaaeqabaqabeGadaaakeaacqWGZbWCcqWGJbWycqWGVbWBcqWGYbGCcqWGLbqzdaWgaaWcbaGaemitaWeabeaakmaabmaabaGaemiEaGhacaGLOaGaayzkaaGaeyypa0ZaaabuaeaacyGGLbqzcqGG4baEcqGGWbaCdaWadaqaaiabdggaHjabdYgaSjabdkhaYnaabmaabaGaemiEaGNaeiilaWIaem4zaCgacaGLOaGaayzkaaaacaGLBbGaayzxaaaaleaacqWGNbWzcqGHiiIZcqWGmbataeqaniabggHiLdaaaa@4E87@. Metabolite weighting is incorporated by calculating total association score as scoreL(x)=∑g∈Lwgexp⁡[alr(x,g)]
 MathType@MTEF@5@5@+=feaafiart1ev1aaatCvAUfKttLearuWrP9MDH5MBPbIqV92AaeXatLxBI9gBaebbnrfifHhDYfgasaacH8akY=wiFfYdH8Gipec8Eeeu0xXdbba9frFj0=OqFfea0dXdd9vqai=hGuQ8kuc9pgc9s8qqaq=dirpe0xb9q8qiLsFr0=vr0=vr0dc8meaabaqaciaacaGaaeqabaqabeGadaaakeaacqWGZbWCcqWGJbWycqWGVbWBcqWGYbGCcqWGLbqzdaWgaaWcbaGaemitaWeabeaakmaabmaabaGaemiEaGhacaGLOaGaayzkaaGaeyypa0ZaaabuaeaacqWG3bWDdaWgaaWcbaGaem4zaCgabeaakiGbcwgaLjabcIha4jabcchaWnaadmaabaGaemyyaeMaemiBaWMaemOCai3aaeWaaeaacqWG4baEcqGGSaalcqWGNbWzaiaawIcacaGLPaaaaiaawUfacaGLDbaaaSqaaiabdEgaNjabgIGiolabdYeambqab0GaeyyeIuoaaaa@518B@, where wg=∏mi∈Θ1Npairsmi
 MathType@MTEF@5@5@+=feaafiart1ev1aaatCvAUfKttLearuWrP9MDH5MBPbIqV92AaeXatLxBI9gBaebbnrfifHhDYfgasaacH8akY=wiFfYdH8Gipec8Eeeu0xXdbba9frFj0=OqFfea0dXdd9vqai=hGuQ8kuc9pgc9s8qqaq=dirpe0xb9q8qiLsFr0=vr0=vr0dc8meaabaqaciaacaGaaeqabaqabeGadaaakeaacqWG3bWDdaWgaaWcbaGaem4zaCgabeaakiabg2da9maarafabaWaaSaaaeaacqaIXaqmaeaacqWGobGtdaqhaaWcbaGaemiCaaNaemyyaeMaemyAaKMaemOCaiNaem4CamhabaGaemyBa02aaSbaaWqaaiabdMgaPbqabaaaaaaaaSqaaiabd2gaTnaaBaaameaacqWGPbqAaeqaaSGaeyicI4SaeuiMdefabeqdcqGHpis1aaaa@44E1@, *m*_*i *_is the *i*^th ^metabolite in the shortest path Θ connecting neighborhood gene *g *with the missing enzyme. Ppairsmi
 MathType@MTEF@5@5@+=feaafiart1ev1aaatCvAUfKttLearuWrP9MDH5MBPbIqV92AaeXatLxBI9gBaebbnrfifHhDYfgasaacH8akY=wiFfYdH8Gipec8Eeeu0xXdbba9frFj0=OqFfea0dXdd9vqai=hGuQ8kuc9pgc9s8qqaq=dirpe0xb9q8qiLsFr0=vr0=vr0dc8meaabaqaciaacaGaaeqabaqabeGadaaakeaacqWGqbaudaqhaaWcbaGaemiCaaNaemyyaeMaemyAaKMaemOCaiNaem4CamhabaGaemyBa02aaSbaaWqaaiabdMgaPbqabaaaaaaa@37D8@ is the total number of gene pairs connected by a metabolite *m*_*i*_. If more than one metabolite connects genes along the path Θ, a metabolite with the smallest *N*_*pairs *_is used.

### Direct likelihood-ratio predictor method

The placement algorithm considers each candidate gene by evaluating *P*(*M*|*D*), which is the conditional probability that a given candidate encodes the desired enzyme (model, *M*) given all available evidence (data, *D *– a set of layer association scores based on different types of association evidence). Following Bayes rule we can calculate that probability (up to a constant) using P(M|D)αP(D|M)P(D)
 MathType@MTEF@5@5@+=feaafiart1ev1aaatCvAUfKttLearuWrP9MDH5MBPbIqV92AaeXatLxBI9gBaebbnrfifHhDYfgasaacH8akY=wiFfYdH8Gipec8Eeeu0xXdbba9frFj0=OqFfea0dXdd9vqai=hGuQ8kuc9pgc9s8qqaq=dirpe0xb9q8qiLsFr0=vr0=vr0dc8meaabaqaciaacaGaaeqabaqabeGadaaakeaacqWGqbaudaqadaqaaiabd2eanjabcYha8jabdseaebGaayjkaiaawMcaaiabeg7aHnaalaaabaGaemiuaa1aaeWaaeaacqWGebarcqGG8baFcqWGnbqtaiaawIcacaGLPaaaaeaacqWGqbaudaqadaqaaiabdseaebGaayjkaiaawMcaaaaaaaa@3EEA@, where *P*(*D*|*M*) is the probability of observing existing associating evidence for a true enzyme-encoding gene. Assuming that different types of associative evidence scores are independent, we calculate probabilities as P(D)=∏ePe(De)
 MathType@MTEF@5@5@+=feaafiart1ev1aaatCvAUfKttLearuWrP9MDH5MBPbIqV92AaeXatLxBI9gBaebbnrfifHhDYfgasaacH8akY=wiFfYdH8Gipec8Eeeu0xXdbba9frFj0=OqFfea0dXdd9vqai=hGuQ8kuc9pgc9s8qqaq=dirpe0xb9q8qiLsFr0=vr0=vr0dc8meaabaqaciaacaGaaeqabaqabeGadaaakeaacqWGqbaudaqadaqaaiabdseaebGaayjkaiaawMcaaiabg2da9maarafabaGaemiuaa1aaSbaaSqaaiabdwgaLbqabaGcdaqadaqaaiabdseaenaaBaaaleaacqWGLbqzaeqaaaGccaGLOaGaayzkaaaaleaacqWGLbqzaeqaniabg+Givdaaaa@3BAF@, where *P*_*e*_(*D*_*e*_) corresponds to the posterior of evidence type *e*. The problem is therefore transformed into estimating tails of association score probability distributions over all genes, and enzyme-encoding genes. For association scores derived for different types of associating evidence and neighborhood layers these probabilities were evaluated empirically from the gene counts, assuming that the likelihood of association increases monotonically with the absolute value of the score.

The self-rank evaluations of known *E. coli *and *S. cerevisiae *metabolic enzyme-encoding genes (see Self-rank validation section in Methods) were performed using leave-one-out validation strategy. In other words, in each case, scores of the candidate being evaluated are not included when calculating *P*(*M*|*D*).

### Alternating decision tree predictor

The *mljava *implementation of the *AdaBoost *algorithm [51] was used to build ADT classifiers based on a set of descriptors, corresponding to different association scores with individual layers of the metabolic network neighborhood. The results presented in Figures 3 and 4 are based on 10-fold validation, 100 iterations of boosting. The training sets included data on only 60% of the true negative (non-metabolic) genes in order to minimize computational time. The candidate genes were prioritized according to the value of the classification margin.

### Predictions with combined association evidence

The self-rank performance illustrated in Figures 3 and 4 was calculated based on candidate association with first three layers of metabolic network neighborhood (neighborhood radius = 3). Association with respect to each layer was described by a separate association score. The predictions were performed using association score ranks: given a candidate gene *x *for a missing enzyme *e*, the value of a descriptor was calculated as a rank of *score*_*L*_(*x*) in a set of scores *S *= {*score*_*L*_(*y*)}_*y*∈*C*_, where *C *is a set of all candidates for a missing enzyme *e*, with higher ranks corresponding to stronger associations. A list of association scores used in combined evidence prediction for *E. coli *and *S. cerevisiae *metabolic models is given in Table 1.

## Authors' contributions

PK carried out calculations of functional associations and enzyme-encoding gene predictions, designed the study, and drafted the manuscript. LC participated in calculations of protein fusion associations. YF participated in development of ADT predictor. DV participated in the design of the study. GMC participated in the design of the study and drafting of the manuscript. All authors read and approved the final manuscript.

## Supplementary Material

Additional File 1 Performance of different profile similarity measures. Click here for file

Additional File 2 Distribution of the number of orthologs per organism. Click here for file

Additional File 3 Performance of different profile similarity measures. Click here for file

Additional File 4 Paralogs and orthologs among metabolic enzymes. Click here for file

Additional File 5 Performance bias due to paralogous metabolic enzymes. Click here for file

Additional File 6 Ortholog co-expression performance. Click here for file

Additional File 7 Performance of protein fusion associations. Click here for file

Additional File 8 Self-rank performance of phenotypic profiles. Click here for file

Additional File 9 Effects of metabolite weighting and association-rank rescaling corrections. Click here for file

Additional File 10 Alternating decision trees and related structures. Click here for file

Additional File 11 Overlap in predictions based on different types of association evidence. Click here for file

Additional File 12 Performance of predictions based on KEGG pathway membership. Click here for file

Additional File 13 Sensitivity of prediction performance on the choice of excluded metabolites. Click here for file

Additional File 15 Prediction performance with and without paralog exclusion. Click here for file

Additional File 16 Gene coverage of different orthology datasets. Additional datasets, including pair-wise functional association matrices for different types of evidence and BLAST-based orthology datasets, are available on the authors' web site[63]. Click here for file

Additional File 14 Chromosome clustering using Gene Order vs. Gene Nucleotide Position. Click here for file

Additional File 17 predictions.zip. Sample predictions of *E. coli *orphans. Additional datasets, including pair-wise functional association matrices for different types of evidence and BLAST-based orthology datasets, are available on the authors' web site[63]. Click here for file

## Figures and Tables

**Table 1:** Association scores used in self-rank tests on combined evidence

Evidence type\Organism	*E.coli*	*S.cerevisiae*
Phylogenetic profile co-occurrence.	• BLAST-based dataset score • KEGG-based dataset score Pairwise associations were calculated using extended hypergeometric and folding corrections, on orthologs established by best bi-directional homology relationship.	
Clustering of genes on the chromosome	• Gene clustering scores. Pairwise associations were calculated on 108 genomes, with KEGG-based orthology dataset	
Gene co-expression	• *E. coli *SMD expression dataset score • Expression of *E. coli *orthologs in *S. cerevisiae *Rosetta dataset.	• *S. cerevisiae *Rosetta expression dataset score • Expression of *S. cerevisiae *orthologs in *E. coli *SMD dataset.
Protein fusion	Separate scores were calculated for different values of *E*_*threshold*_: • 10^-2^ • 10^-5^ • 10^-10^	Separate scores were calculated for different values of *E*_*threshold*_: • 10^-3^ • 10^-5^ • 10^-10^
Protein interactions		• Interaction score based on PIE dataset
