# Supplementary material for: Identifying metabolic enzymes with multiple types of association evidence
Source: BMC Bioinformatics. 2006 Mar 29;7:177. doi: 10.1186/1471-2105-7-177 (PMC1450304; doi:10.1186/1471-2105-7-177)
Supplement: Additional File 1 — Performance of different profile similarity measures. [file 1471-2105-7-177-S1.pdf]

Figure 1.

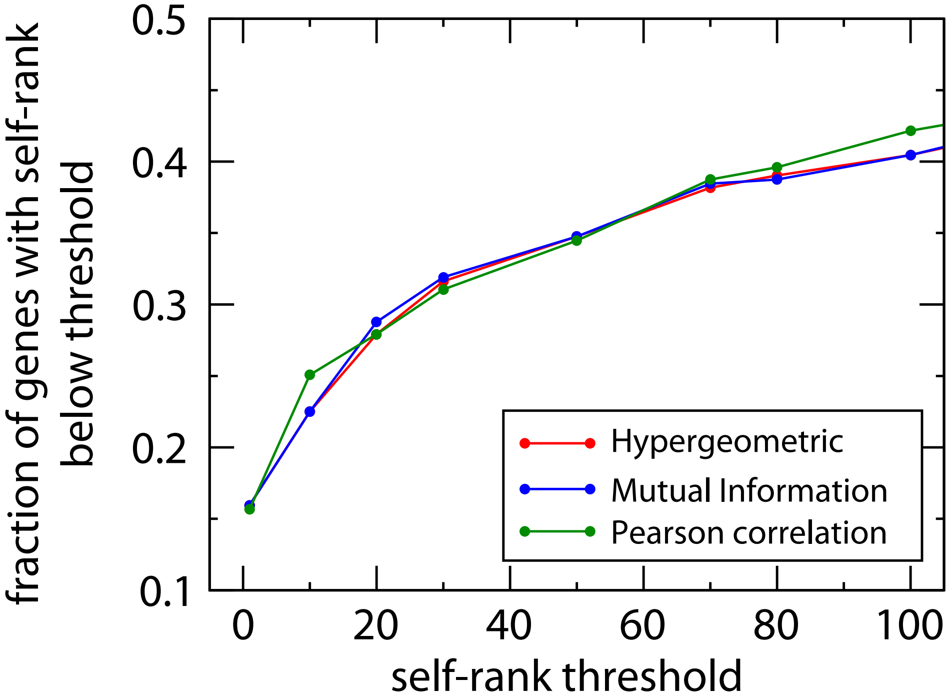

Performance of different profile similarity measures. *E. coli* self-rank performance based on the phylogenetic profile associations with the first layer of the neighborhood is shown for scores calculated using Hypergeometric probability, mutual information and Pearson linear correlation. BLAST-based orthology dataset was used.
