# Supplementary material for: Identifying metabolic enzymes with multiple types of association evidence
Source: BMC Bioinformatics. 2006 Mar 29;7:177. doi: 10.1186/1471-2105-7-177 (PMC1450304; doi:10.1186/1471-2105-7-177)
Supplement: Additional File 2 — Distribution of the number of orthologs per organism. [file 1471-2105-7-177-S2.pdf]

Figure 2.

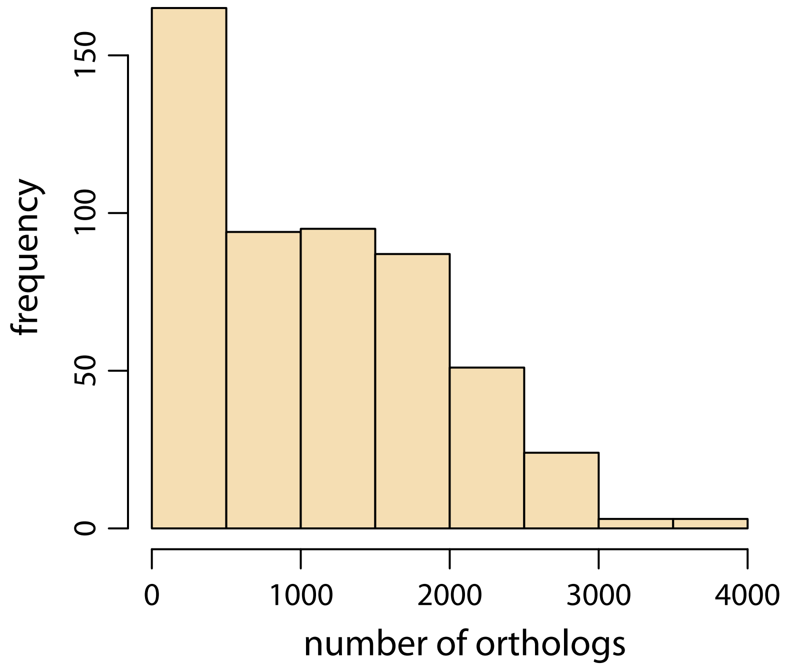

Distribution of the number of orthologs per organism. Distribution of the number of orthologs of *E. coli* genes is shown for the organisms included in the BLAST-based orthology dataset. Organisms containing orthologs for fewer than 4% of *E. coli* genes were excluded.
