# Supplementary material for: Identifying metabolic enzymes with multiple types of association evidence
Source: BMC Bioinformatics. 2006 Mar 29;7:177. doi: 10.1186/1471-2105-7-177 (PMC1450304; doi:10.1186/1471-2105-7-177)
Supplement: Additional File 3 — Performance of different profile similarity measures. [file 1471-2105-7-177-S3.pdf]

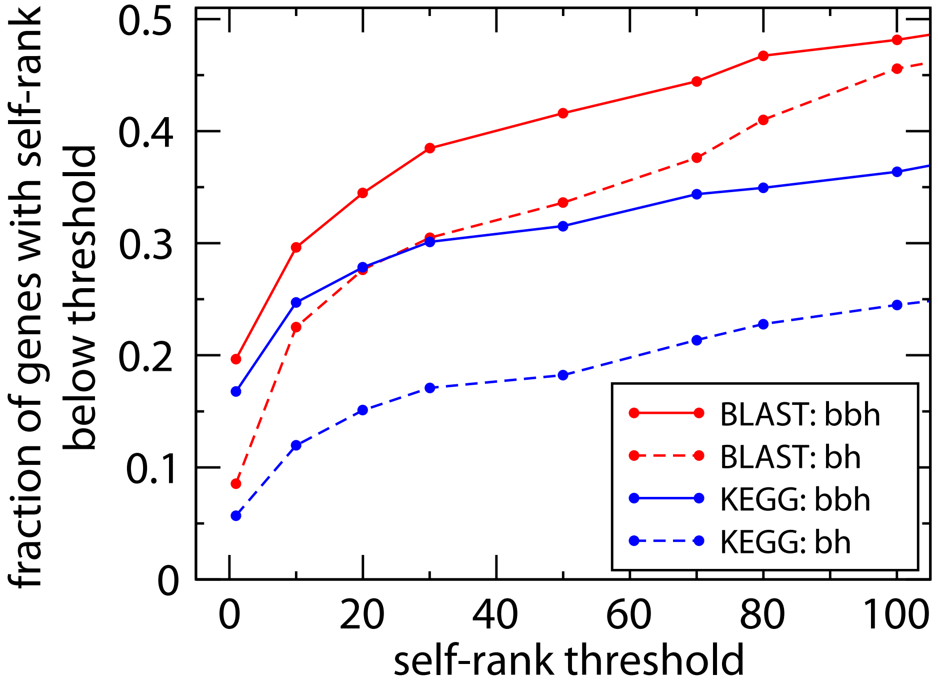

Best homologs and bi-directional best homologs. *E. coli* self-rank performance based on the phylogenetic profile associations with the first layer of the neighborhood is shown for different orthology datasets. The performance is shown for BLAST-based and KEGG-based datasets constructed using closest homologs (bh), and best bi-directional homologs (bbh). Phylogenetic profile association was calculated using extended hypergeometric distribution with folding.
