# Supplementary material for: Identifying metabolic enzymes with multiple types of association evidence
Source: BMC Bioinformatics. 2006 Mar 29;7:177. doi: 10.1186/1471-2105-7-177 (PMC1450304; doi:10.1186/1471-2105-7-177)
Supplement: Additional File 4 — Paralogs and orthologs among metabolic enzymes. [file 1471-2105-7-177-S4.pdf]

Figure 4.

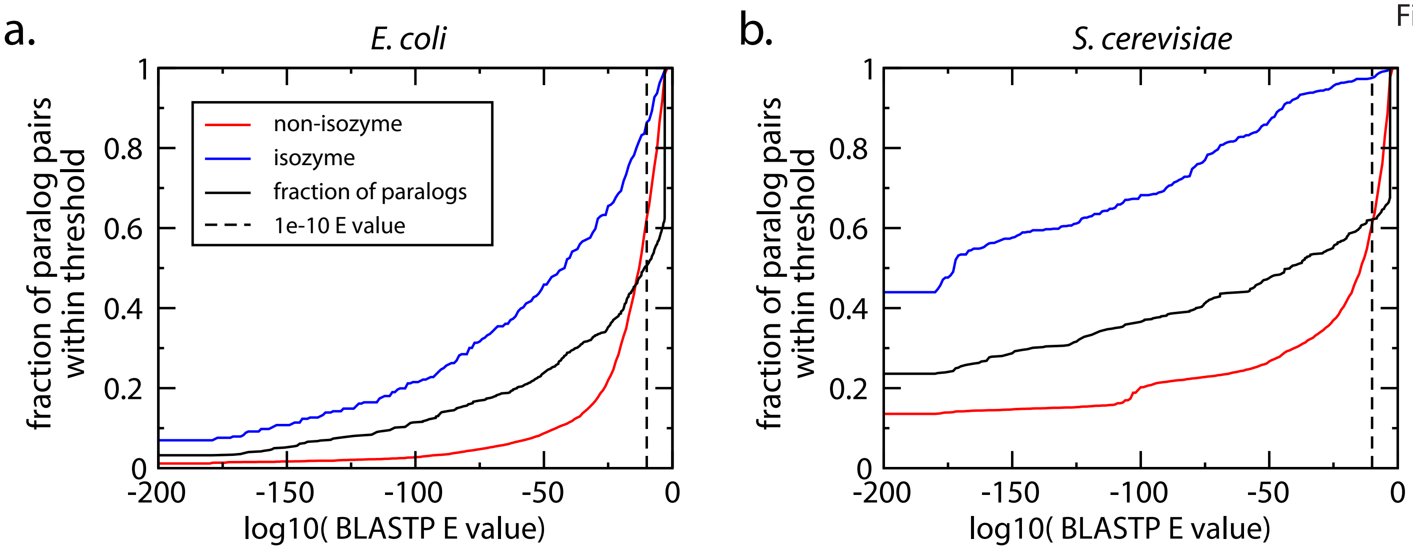

Paralogs and orthologs among metabolic enzymes. Fraction of homologous enzyme pairs is shown as a function of the homology threshold (BLASTp E value) for **a.** *E. coli* and **b.** *S. cerevisiae* metabolic models. Distribtion is shown separately for pairs of enzymes catalyzing same metabolic reaction (isozymes, shown in blue), and all other enzyme pairs (shown in red).

In addition to pair statistics, plots show the fraction of metabolic enzymes that are paralogs of some other metabolic enzyme (black line). The results presented in the manuscript exclude self-ranks of metabolic enzymes that have sequence homology E value below 1e-10 with respect to any other metabolic enzyme (vertical dashed line).
