# Supplementary material for: Identifying metabolic enzymes with multiple types of association evidence
Source: BMC Bioinformatics. 2006 Mar 29;7:177. doi: 10.1186/1471-2105-7-177 (PMC1450304; doi:10.1186/1471-2105-7-177)
Supplement: Additional File 5 — Performance bias due to paralogous metabolic enzymes. [file 1471-2105-7-177-S5.pdf]

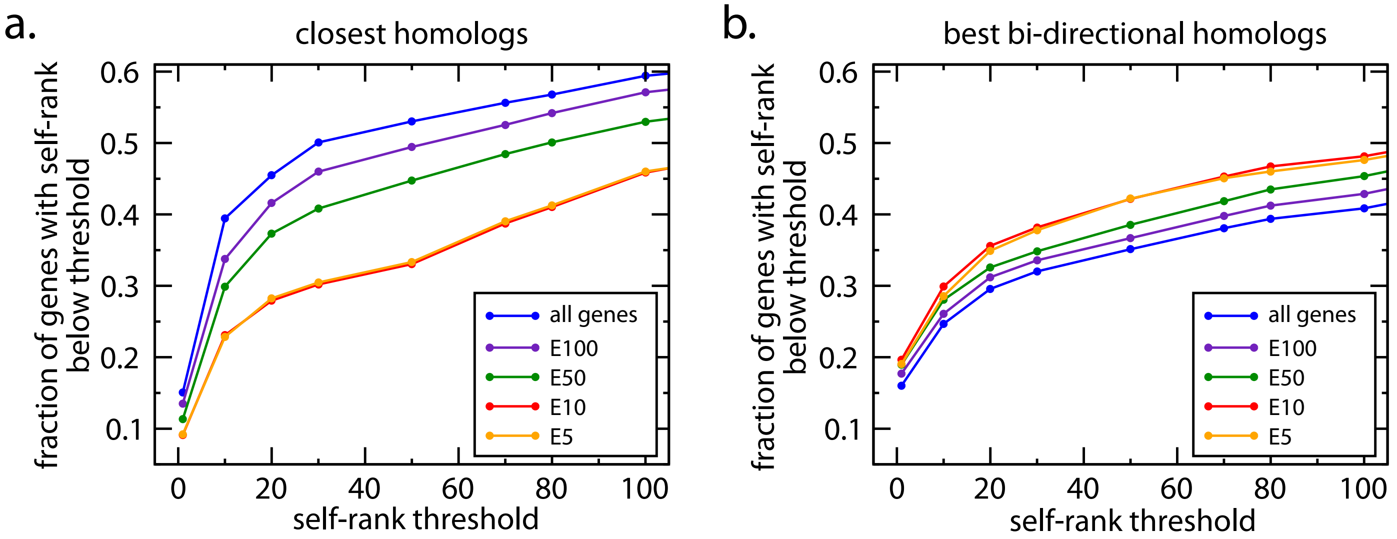

Performance bias due to paralogous metabolic enzymes. Self-rank performance based on the phylogenetic profile associations with the first layer of the neighborhood is shown for all enzymes of E. coli metabolism (all genes), and for the set of metabolic enzymes that excludes homologous enzymes, as determined by BLASTp E value thresholds of 10^{-5}, 10^{-10}, 10^{-50} and 10^{-100}. The performance is shown for BLAST orthology dataset based on **a.** closest homologs, and **b.** on best bi-directional homologs.

Inclusion of homologous enzymes results in an increased number of identical or overlapping orthology mappings in the "closest homologs" dataset (**a.**), resulting in a significant overestimation of the performance due to indirect homology predictions. By contrast, in the case of the "best bi-directional" orthology dataset (**b.**), inclusion of homologous enzymes reduces the number of orthology information, resulting in underestimation of performance.
