# Supplementary material for: Identifying metabolic enzymes with multiple types of association evidence
Source: BMC Bioinformatics. 2006 Mar 29;7:177. doi: 10.1186/1471-2105-7-177 (PMC1450304; doi:10.1186/1471-2105-7-177)
Supplement: Additional File 6 — Ortholog co-expression performance. [file 1471-2105-7-177-S6.pdf]

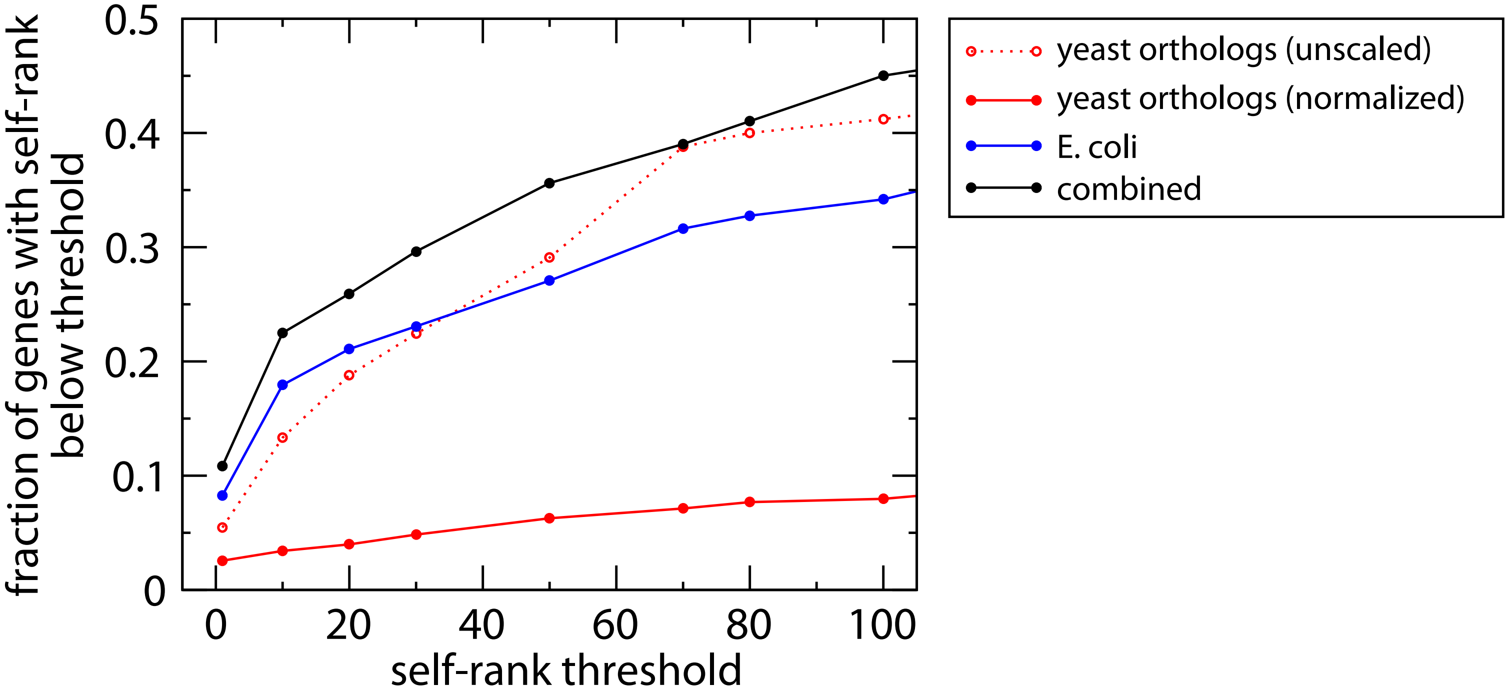

Ortholog co-expression performance. *E. coli* self-rank performance is shown separately using mRNA expression data from *E. coli* microarray studies (E. coli), using expression of *S. cerevisiae* orthologs of *E. coli* genes in the Rosetta compendium dataset (yeast orthologs), and for the combination of the two association scores (combined). Performance is shown on the set of metabolic genes with orthologs in *S. cerevisiae* (unscaled), and also normalized to the total number of known *E. coli* metabolic genes (normalized). Prediction were generated using using ADT classifer (with 10 fold validation) based on association scores to 3 layers of the metabolic network neighborhood.
