# Supplementary material for: Identifying metabolic enzymes with multiple types of association evidence
Source: BMC Bioinformatics. 2006 Mar 29;7:177. doi: 10.1186/1471-2105-7-177 (PMC1450304; doi:10.1186/1471-2105-7-177)
Supplement: Additional File 7 — Performance of protein fusion associations. [file 1471-2105-7-177-S7.pdf]

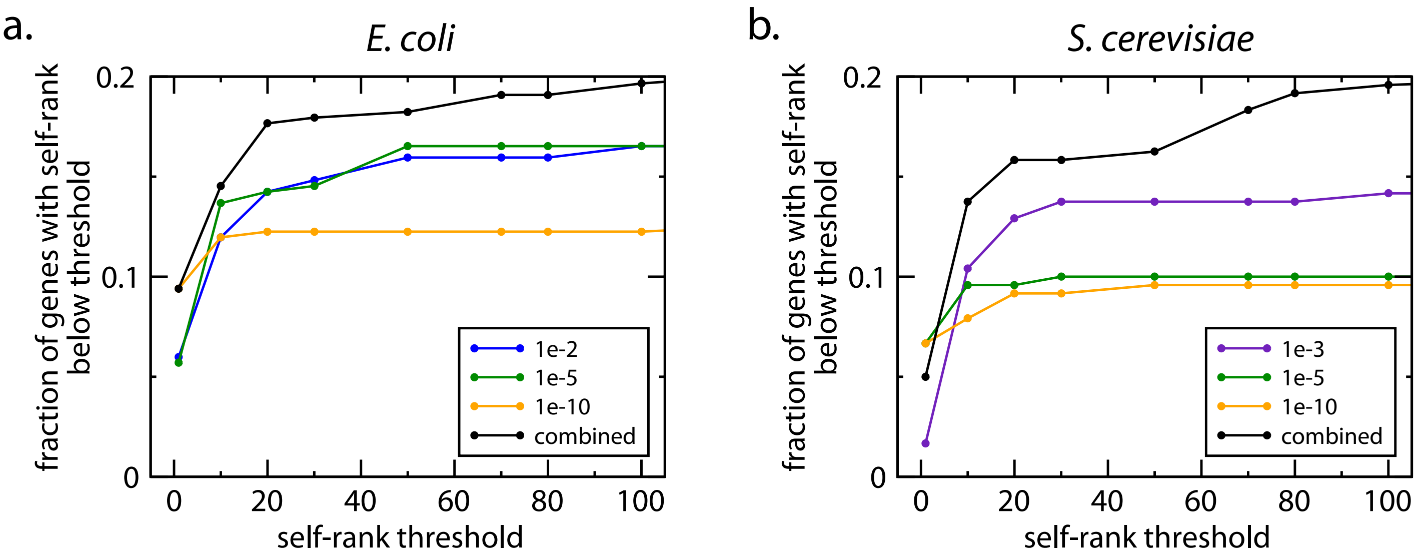

Performance of protein fusion associations. Self-rank performance based on fusion scores is shown for **a.** *E. coli* and **b.** *S. cerevisiae* metabolic enzymes. The performance is shown for fusion data based on different E value thresholds (10^{-2}, 10^{-5},10^{-10} for *E. coli*; 10^{-3}, 10^{-5},10^{-10} for *S. cerevisiae*) and their combination. The predictions are calculated using ADT classifer based on association with 3 layers of the metabolic network neighborhood with 10-fold validation.
