# Supplementary material for: Identifying metabolic enzymes with multiple types of association evidence
Source: BMC Bioinformatics. 2006 Mar 29;7:177. doi: 10.1186/1471-2105-7-177 (PMC1450304; doi:10.1186/1471-2105-7-177)
Supplement: Additional File 8 — Self-rank performance of phenotypic profiles. [file 1471-2105-7-177-S8.pdf]

Figure 8.

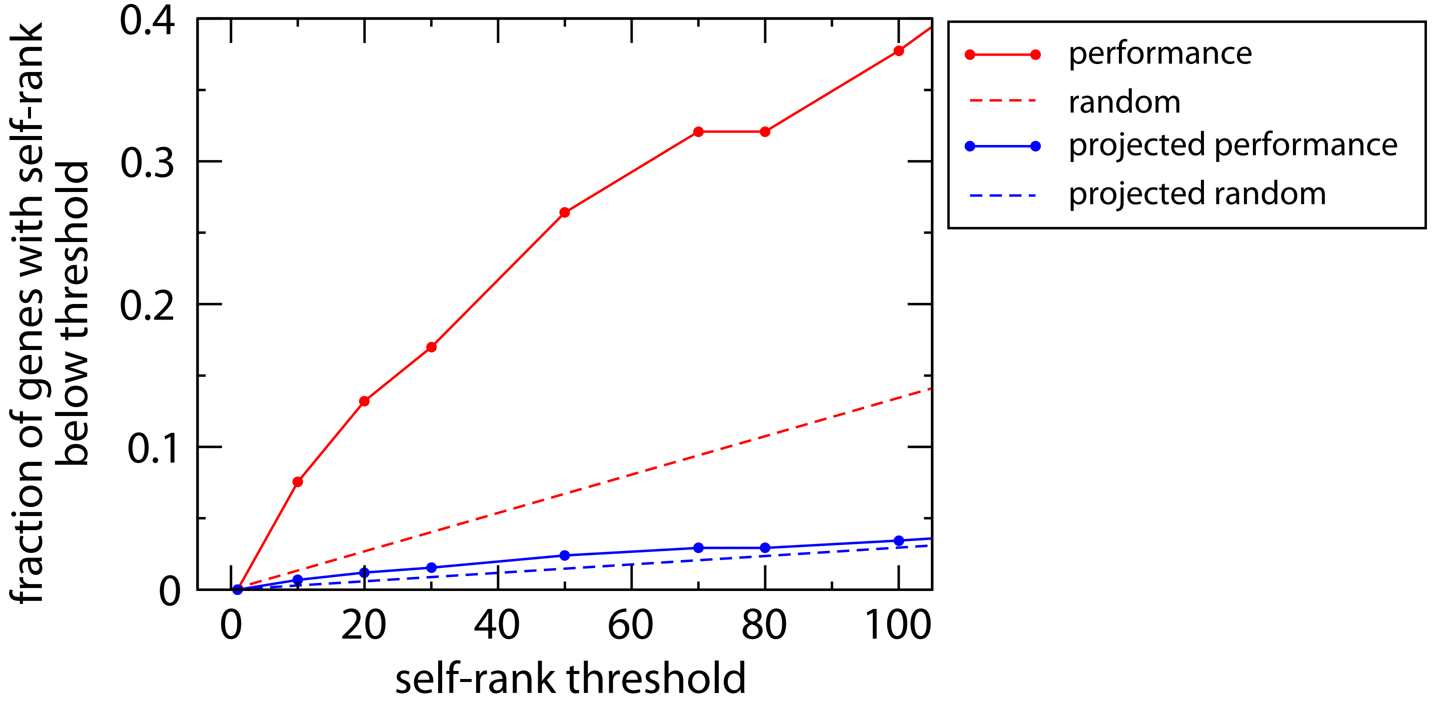

Self-rank performance of phenotypic profiles. *S. cerevisiae* self-rank performance based on similarity of phenotypic profiles is shown for the set of genes for which phenotypic data is available (red). Overall, normalized performanced on a set of all metabolic enzymes is estimated (blue). Unlike all other presented results, the set of metabolic enzymes was not filtered to exclude homologous enzyme pairs. Performance of a completely noninformative (random) scores is shown by the dashed lines for each case. Predictions were generated based on association scores with 3 layers of the metabolic network neighborhood, combined using ADT classifier with 10 fold validation.
