# Supplementary material for: Identifying metabolic enzymes with multiple types of association evidence
Source: BMC Bioinformatics. 2006 Mar 29;7:177. doi: 10.1186/1471-2105-7-177 (PMC1450304; doi:10.1186/1471-2105-7-177)
Supplement: Additional File 9 — Effects of metabolite weighting and association-rank rescaling corrections. [file 1471-2105-7-177-S9.pdf]

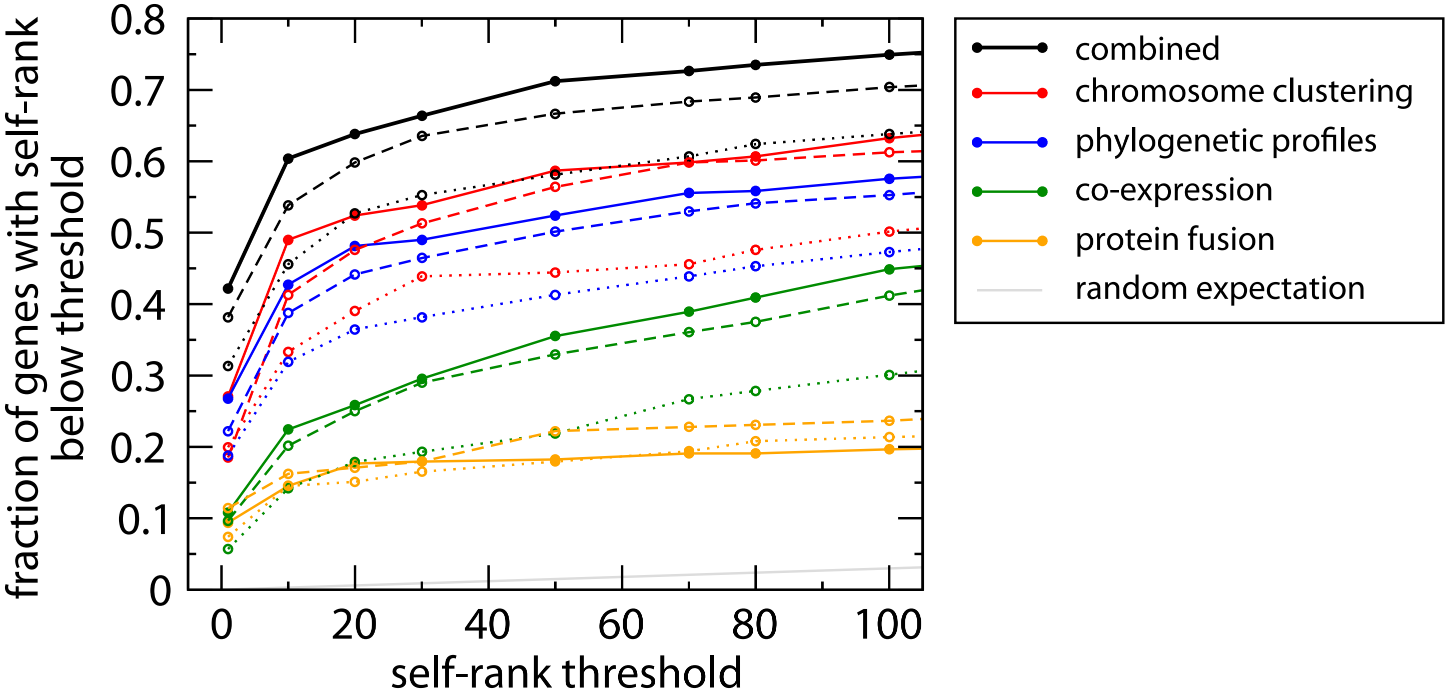

Effects of metabolite weighting and association-rank rescaling corrections. *E. coli* self-rank performance is shown for individual types of association evidence and for their combination. Performance based on metabolite weighting and association-rank rescaling corrections is shown in solid lines, with association-rank correction only in dashed lines, and without either correction in dotted lines. Prediction were generated using using ADT classifer (with 10 fold validation) based on association scores to 3 layers of the metabolic network neighborhood.
