# Supplementary material for: Identifying metabolic enzymes with multiple types of association evidence
Source: BMC Bioinformatics. 2006 Mar 29;7:177. doi: 10.1186/1471-2105-7-177 (PMC1450304; doi:10.1186/1471-2105-7-177)
Supplement: Additional File 10 — Alternating decision trees and related structures. [file 1471-2105-7-177-S10.pdf]

Figure 10.

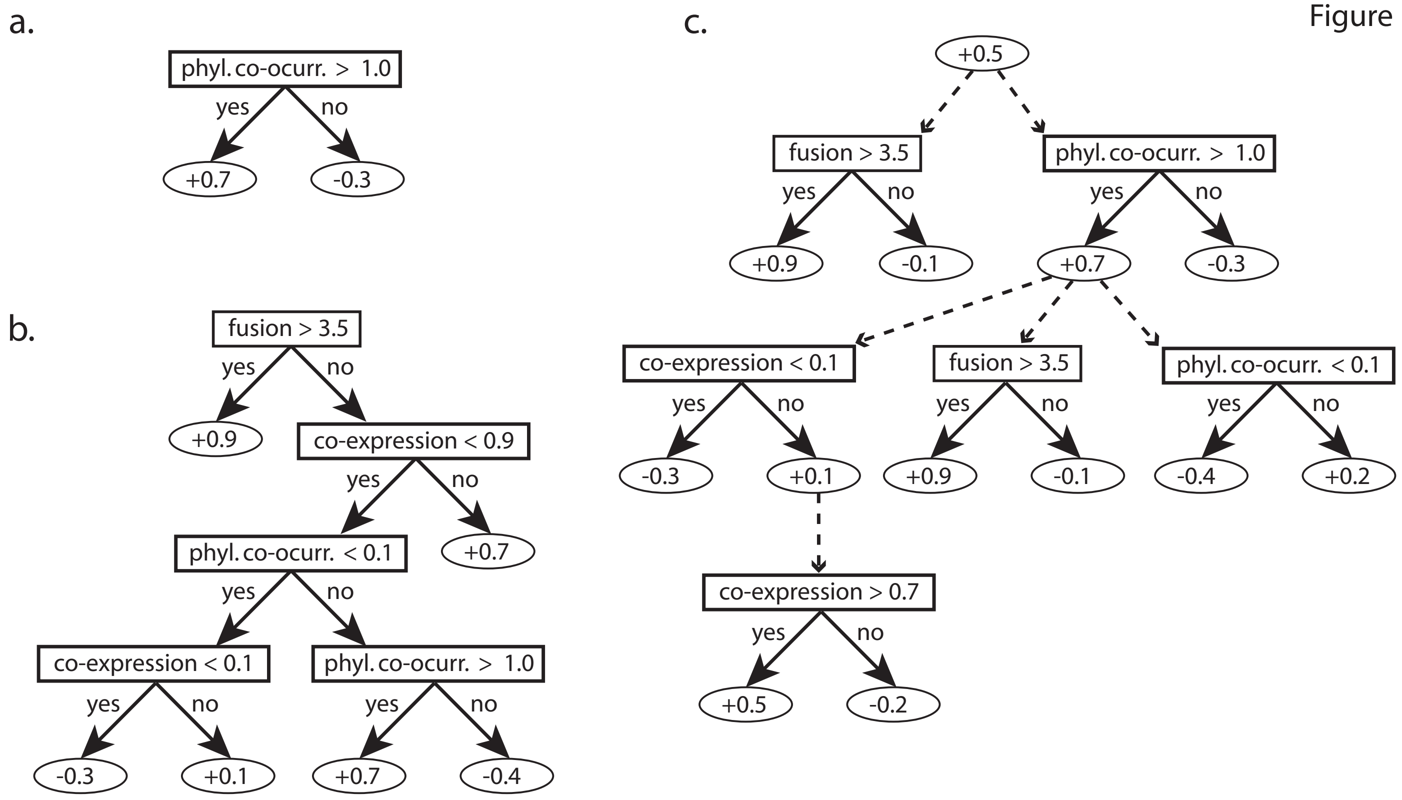

Alternating decision trees and related structures. a. An example of a decision stump classifying based on a value of a single variable (phylogenetic co-ocurrence). The decision node (or splitter node) is drawn as a rectangle, and the prediction nodes as ovals. Classification margin values are shown within the prediction nodes. **b.** A classic decision tree structure, sequentially classifying based on several variables. **c.** Alternating decision tree based on multiple variables, consisting of alternating decision and prediction layers. More than one decision node can be attached to each prediction node. The overall classification margin is determined by summing prediction node values along all valid classification paths.
