# Supplementary material for: Identifying metabolic enzymes with multiple types of association evidence
Source: BMC Bioinformatics. 2006 Mar 29;7:177. doi: 10.1186/1471-2105-7-177 (PMC1450304; doi:10.1186/1471-2105-7-177)
Supplement: Additional File 11 — Overlap in predictions based on different types of association evidence. [file 1471-2105-7-177-S11.pdf]

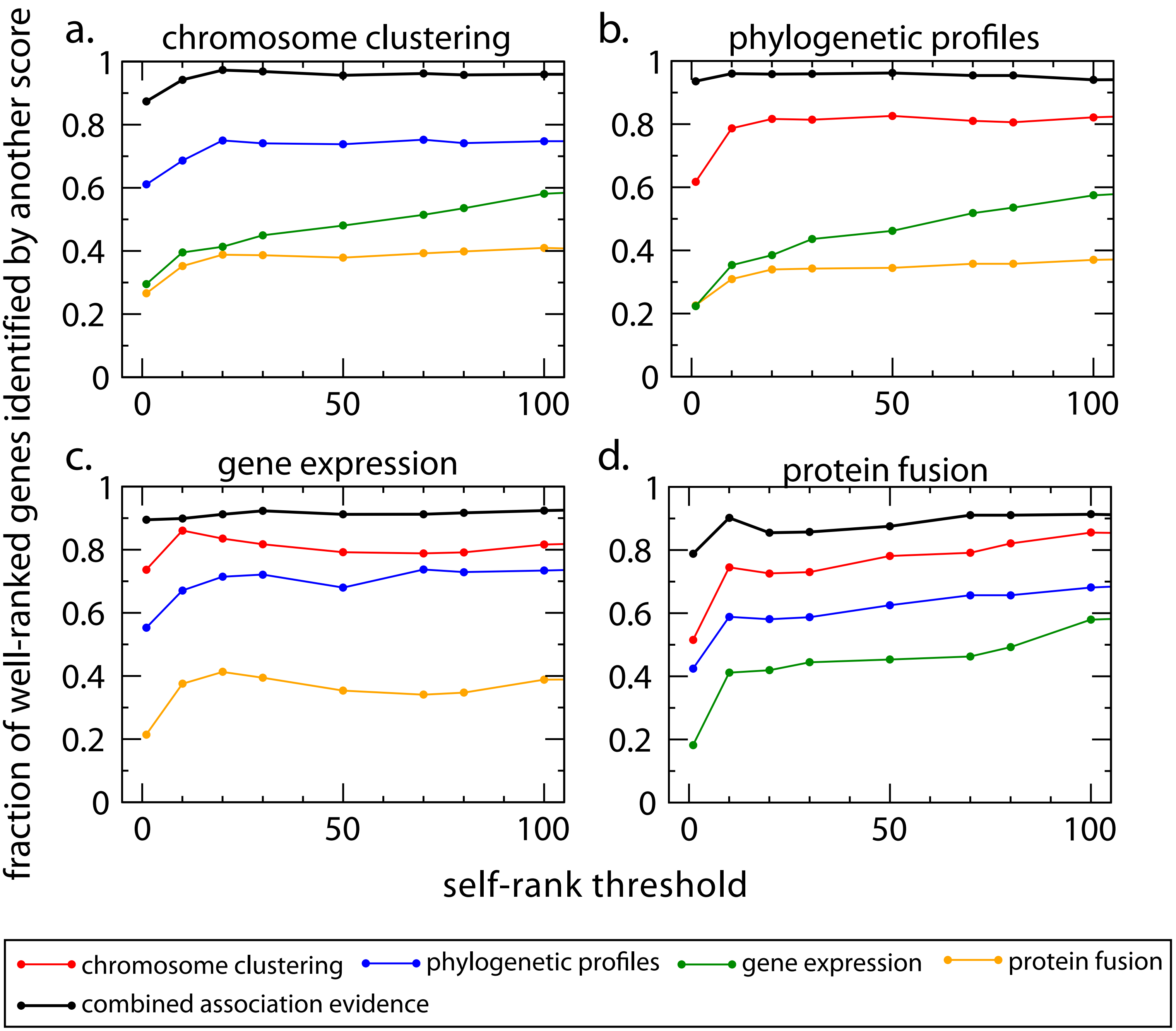

Overlap in predictions of different types of association evidence. **a.** Fraction of metabolic enzymes predicted based chromosome clustering association score that are also predicted based on other associating evidence: phylogenetic profiles (blue), gene expression (green) and protein fusion (orange). Fractions for different self-rank thresholds are shown. Analogous fractional prediction coverage overlap is shown for predictions based on **b.** phylogenetic profiles, **c.** gene expression and **d.** protein fusion. All predictions are generated as specified in Figure 3 of the main manuscript.
