# Supplementary material for: Identifying metabolic enzymes with multiple types of association evidence
Source: BMC Bioinformatics. 2006 Mar 29;7:177. doi: 10.1186/1471-2105-7-177 (PMC1450304; doi:10.1186/1471-2105-7-177)
Supplement: Additional File 12 — Performance of predictions based on KEGG pathway membership. [file 1471-2105-7-177-S12.pdf]

Figure 12.

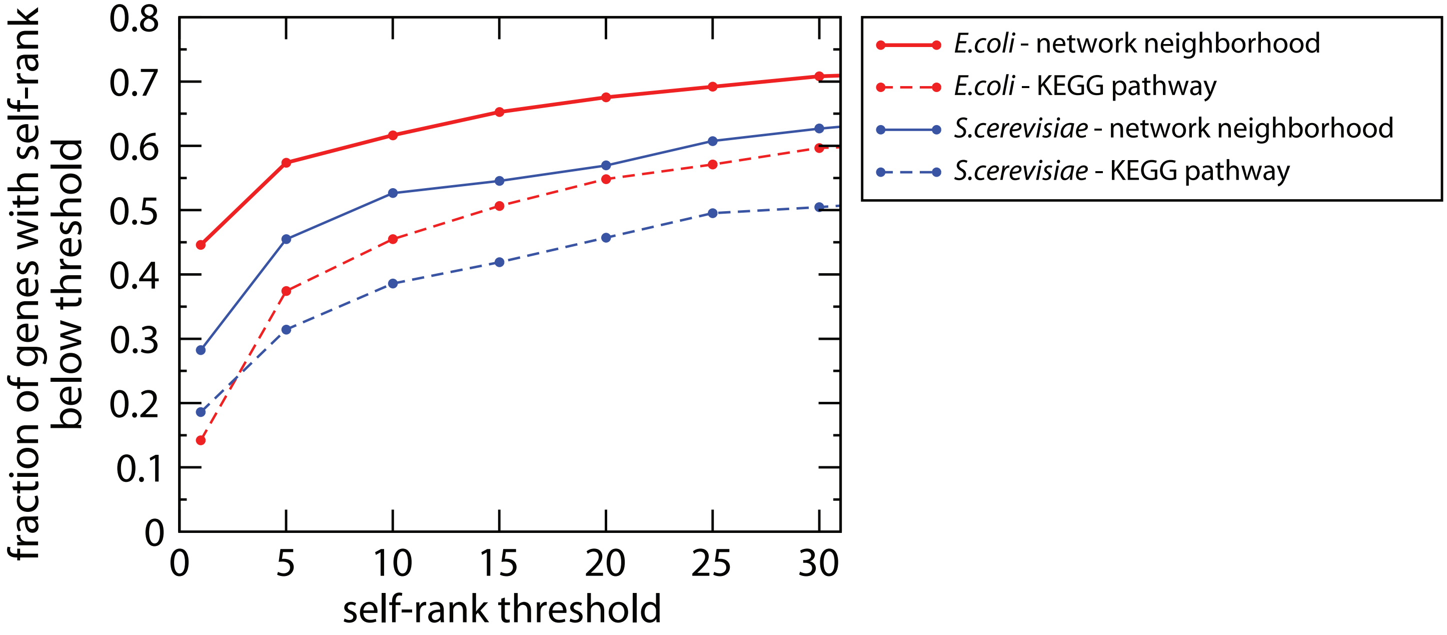

Performance of predictions based on KEGG pathway membership. Fraction of metabolic enzymes predicted is compared for predictions made using regular metabolic network neighborhood (as defined in the manuscript, solid lines), and KEGG pathway neighborhoods (dashed lines). Predictions are made using combined association evidence, DLR method, without metabolite weighting. *E.coli* performance is shown in red, *S.cerevisiae* performance is shown in blue. KEGG pathway neighborhoods consist of a single layer, which includes all enzyme-encoding genes of the target organism (*E.coli* or *S.cerevisiae*) that appear in the same pathway as the enzyme being tested. The test set of known *E. coli* metabolic enzymes (see Methods) was reduced to include only those metabolic enzymes which are included in KEGG pathway maps.
