# Supplementary material for: Identifying metabolic enzymes with multiple types of association evidence
Source: BMC Bioinformatics. 2006 Mar 29;7:177. doi: 10.1186/1471-2105-7-177 (PMC1450304; doi:10.1186/1471-2105-7-177)
Supplement: Additional File 13 — Sensitivity of prediction performance on the choice of excluded metabolites. [file 1471-2105-7-177-S13.pdf]

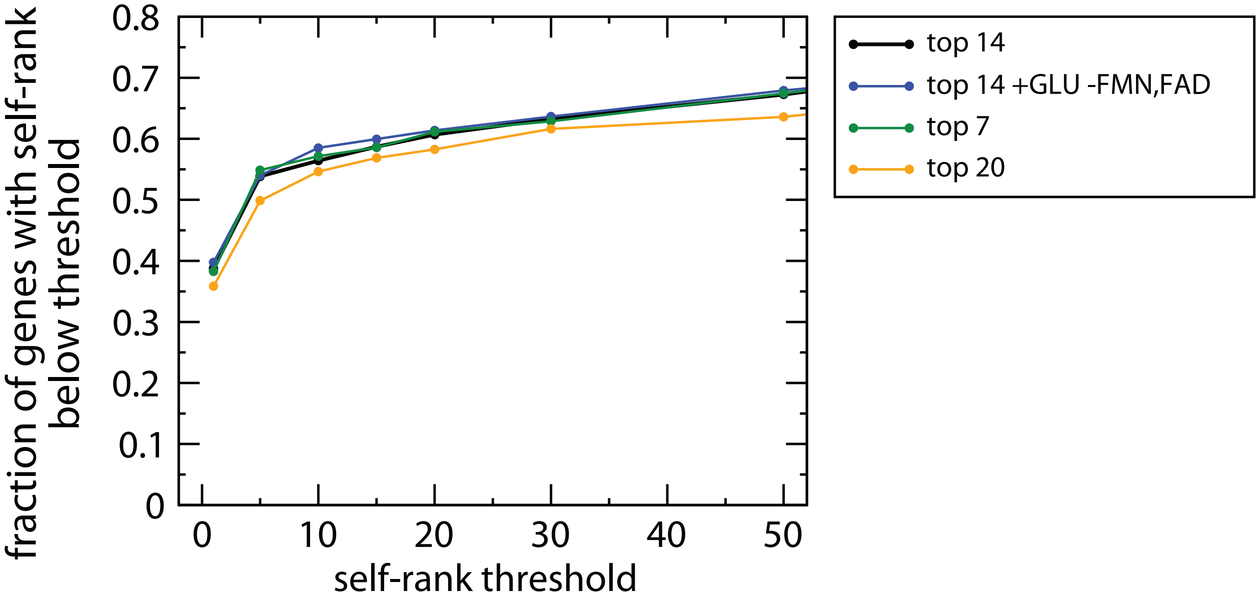

Sensitivity of prediction performance on the choice of excluded metabolites. Fraction of *E.coli* metabolic enzymes predicted is shown for different sets of excluded metabolites. "top14" (black) excludes connections established by top 14 most common metabolites, and is used for all other results presented in the manuscript. "top14+GLU-FNM,FAD" (blue) excludes connections established by top 14 most common metabolites and by FMN, FMNH2, FAD, FADH2, but includes connections established by glutamate. "top 7" (green) and "top 20" (orange) exclude connections established by top 7 and top 20 most common metabolites respectfully. Predictions are generated using DLR method with combined association evidence. The results illustrate low sensitivity of the overall algorithm performance to the exact choice of the excluded metabolite set.
