# Supplementary material for: Identifying metabolic enzymes with multiple types of association evidence
Source: BMC Bioinformatics. 2006 Mar 29;7:177. doi: 10.1186/1471-2105-7-177 (PMC1450304; doi:10.1186/1471-2105-7-177)
Supplement: Additional File 15 — Prediction performance with and without paralog exclusion. [file 1471-2105-7-177-S15.pdf]

Figure 15.

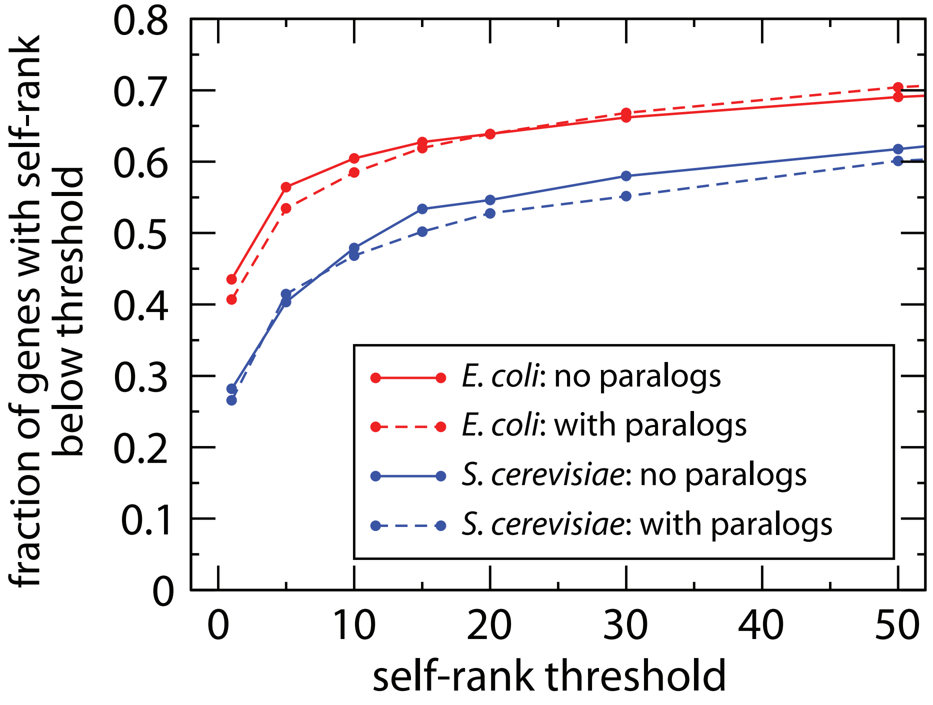


**Prediction performance with and without paralog exclusion.** Fraction of enzymes predicted within different self-rank thresholds is shown for *E. coli* (red) and *S. cerevisiae* (blue) metabolic models. For each organism, performance is shown for two different enzyme-encoding gene test sets: a set of all valid enzyme-encoding genes (dashed lines; 612 genes for *E. coli*, 504 for *S. cerevisiae*), and a set that excludes all paralogous enzymes (solid lines; 351 for *E. coli*, 240 for *S. cerevisiae*). The paralog filtered set is the default test set used throughout the manuscript (see Methods). To avoid bias from overlapping orthology mappings of known paralogs when calculating performance on a complete test set (with paralogs), phylogenetic profile associations between paralogous gene pairs were omitted in evaluation of candidates. Performance was evaluated DLR method on combined association evidence.
