# Supplementary material for: Identifying metabolic enzymes with multiple types of association evidence
Source: BMC Bioinformatics. 2006 Mar 29;7:177. doi: 10.1186/1471-2105-7-177 (PMC1450304; doi:10.1186/1471-2105-7-177)
Supplement: Additional File 16 — Gene coverage of different orthology datasets. Additional datasets, including pair-wise functional association matrices for different types of evidence and BLAST-based orthology datasets, are available on the authors' web site[63]. [file 1471-2105-7-177-S16.pdf]

	E. coli			*S. cerevisiae*		
	Metabolic	Other	All	Metabolic	Other	All
COG	98.7%	76.8%	81.3%	92.1%	31.3%	46.7%
KEGG	99.7%	97.6%	98.0%	96.8%	88.0%	89.0%
BLAST	98.7%	98.9%	99.2%	98.7%	94.3%	94.9%

Gene coverage of different orthology datasets. The table shows fraction of metabolic, non-metabolic (other) and all genes of *E. coli* and *S. cerevisiae* genomes covered by COGs, KEGG-based orthology dataset (best bi-directional hits) and BLAST-based dataset.
