# Supplementary material for: Identifying metabolic enzymes with multiple types of association evidence
Source: BMC Bioinformatics. 2006 Mar 29;7:177. doi: 10.1186/1471-2105-7-177 (PMC1450304; doi:10.1186/1471-2105-7-177)
Supplement: Additional File 14 — Chromosome clustering using Gene Order vs. Gene Nucleotide Position. [file 1471-2105-7-177-S14.pdf]

Figure 14.

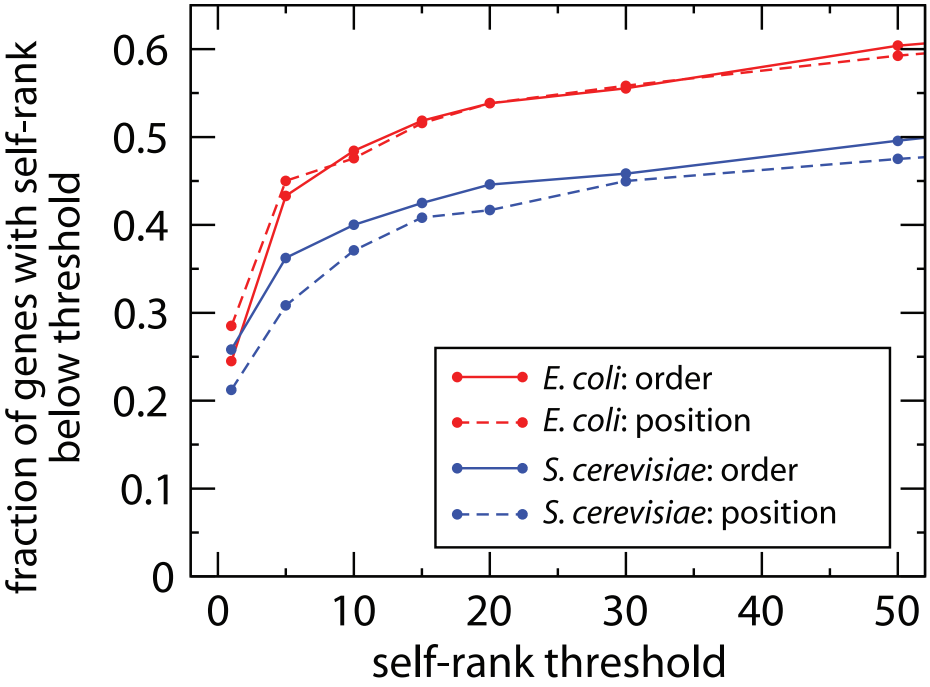

Chromosome clustering using Gene Order vs. Gene Nucleotide Position. Predictive performance of two different types of chromosome clustering scores is shown. Gene order score (solid lines) is calculated based on gene order distance, as described in the manuscript. Position score (dashed lines) is calculated based on the nucleotide distance between genes (see below). Predictions are generated using DLR method.

Position score is calculated using the same approach as gene order score, with the following differences:

1. Null hypothesis is that genes are randomly and uniformly distributed along the entire length of the chromosome.
2. For circular chromosomes, probability $P(d_g(x,y))$ is calculated according to:

$$\begin{cases} P(d_g(x,y)) = p_s & x, y \text{ are on different chromosomes} \\ P(d_g(x,y)) = p_s \dfrac{2d_g(x,y)}{l} & \text{otherwise} \end{cases}$$

where p_s is the probability of two genes occurring on one chromosome, calculated by counting appropriate gene pairs in genome *g*. l is the length of the chromosome.

For linear chromosomes:

$$\begin{cases} P(d_g(x,y)) = p_s & x, y \text{ are on different chromosomes} \\ P(d_g(x,y)) = p_s \left[\dfrac{2d_g(x,y)}{l} - \left(\dfrac{d_g(x,y)}{l} \right)^2 \right] & \text{otherwise} \end{cases}$$
