# Supplementary material for: Identifying metabolic enzymes with multiple types of association evidence
Source: BMC Bioinformatics. 2006 Mar 29;7:177. doi: 10.1186/1471-2105-7-177 (PMC1450304; doi:10.1186/1471-2105-7-177)
Supplement: Additional File 17 — predictions.zip. Sample predictions of E. coli orphans. Additional datasets, including pair-wise functional association matrices for different types of evidence and BLAST-based orthology datasets, are available on the authors' web site[63]. [file 1471-2105-7-177-S17.zip › predictions/Readme.pdf]

Predicted candidates file format

Each file contains entries for the top 500 genes encoding a specified enzymatic function. A sample candidate record is shown below, with further explanations for each marked item.

candidate #1: b1444
(1) **likelihood ratios:** Total 1.1e+11; Clust 19.1,257.8,; BLAST 28.8,4.5,; KEGG 7.2,35.3,; ExprEco 75.7,36.6,; ExprSce ,,; FusE2 1.0,0.9,; FusE5 1.0,0.9,0.7; FusE10 ,0.8,0.6;
gene name: b1444 (2) (NP_415961) **gi:** 16129403 **length:** 474aa
(3) **description:** putative aldehyde dehydrogenase [Escherichia coli K12].
coverage: Archaea[1e-100] Bacteria[0.0] Eukaryota[0.0] (4)
conserved domains: (Evalue, description)
(5) 1e-136 COG1012, PutA, NAD-dependent aldehyde dehydrogenases [Energy production and conversion].
1e-136 pfam00171, Aldedh, Aldehyde dehydrogenase family. This family of dehydrogenases act on aldehyde substrates. Members use NADP as a cofactor. The family includes the following members: The prototypical members are the aldehyde dehydrogenases EC:1.2.1.3. Succinate-semialdehyde dehydrogenase EC:1.2.1.16. Lactaldehyde dehydrogenase EC:1.2.1.22. Benzaldehyde dehydrogenase EC:1.2.1.28. Methylmalonate-semialdehyde dehydrogenase EC:1.2.1.27. Glyceraldehyde-3-phosphate dehydrogenase EC:1.2.1.9. Delta-1-pyrroline-5-carboxylate dehydrogenase EC: 1.5.1.12. Acetaldehyde dehydrogenase EC:1.2.1.10. Glutamate-5-semialdehyde dehydrogenase EC:1.2.1.41. This family also includes omega crystallin, an eye lens protein from squid and octopus that has little aldehyde dehydrogenase activity..
3e-59 COG4230, COG4230, Delta 1-pyrroline-5-carboxylate dehydrogenase [Energy production and conversion].

(1) Combined and individual score likelihood ratios calculated by the DLR method are shown. The ***Total*** value is the multiple of all likelihood ratios which is used to prioritize the candidates. It is followed by ratios stemming from associations with first three layers of the metabolic network neighborhood. ***Clust*** - chromosome clustering associations; ***BLAST*** - phylogenetic profile associations based on BLAST orthology dataset; ***KEGG*** - phylogenetic profile associations based on KEGG orthology dataset; ***ExprEco*** - co-expression based on SMD *E. coli* data; ***ExprSCE*** - co-expression of orthologous genes in *S. cerevisiae*; ***FusE2***, ***FusE5*** and ***FusE10*** - protein fusion associations calculated with three different values of $E_{threshold}$: 1e-2, 1e-5 and 1e-10.

(2) Standard gene name, followed by RefSeq accession number, GenBank Id and length of the aminoacid sequence

(3) Gene description from RefSeq

(4) Phylogenetic coverage. BLASTP E-value is shown for a best homolog in three superkingdoms.

(5) Matching domains from CDD database. An rpsBLAST E-value is shown, together with a description of a matched domain.
